# Peptidomics of enteroendocrine cells and characterisation of potential effects of a novel preprogastrin derived-peptide on glucose tolerance in lean mice

**DOI:** 10.1016/j.peptides.2021.170532

**Published:** 2021-06

**Authors:** Sam G. Galvin, Pierre Larraufie, Richard G. Kay, Haidee Pitt, Elise Bernard, Anne K. McGavigan, Helen Brant, John Hood, Laura Sheldrake, Shannon Conder, Dawn Atherton-Kemp, Van B. Lu, Elisabeth A.A. O’Flaherty, Geoffrey P. Roberts, Carina Ämmälä, Lutz Jermutus, David Baker, Fiona M. Gribble, Frank Reimann

**Affiliations:** a University of Cambridge Metabolic Research Laboratories, WT-MRC Institute of Metabolic Science, Addenbrooke’s Hospital, Hills Road, Cambridge, CB2 0QQ, UK; b Animal Science and Technologies - UK, AstraZeneca, The Babraham Institute, Cambridge, UK; c ADPE, AstraZeneca Ltd, Granta Park, Cambridge, CB21 6GH, UK; d Pharmacokinetics, AstraZeneca Ltd, Granta Park, Cambridge, UK; e Bioscience Metabolism, Research and Early Development Cardiovascular, Renal and Metabolism (CVRM), BioPharmaceuticals R&D, AstraZeneca, Gothenburg, 431 83 Mölndal, Sweden; f Research and Early Development Cardiovascular, Renal and Metabolism (CVRM), BioPharmaceuticals R&D, AstraZeneca Ltd, Cambridge, UK

**Keywords:** Enteroendocrine cells, Peptidomics, Mass spectrometry, Prohormones, Granins, Progastrin

## Abstract

**Objectives:**

To analyse the peptidomics of mouse enteroendocrine cells (EECs) and human gastrointestinal (GI) tissue and identify novel gut derived peptides.

**Methods:**

High resolution nano-flow liquid chromatography mass spectrometry (LC–MS/MS) was performed on (i) flow-cytometry purified NeuroD1 positive cells from mouse and homogenised human intestinal biopsies, (ii) supernatants from primary murine intestinal cultures, (iii) intestinal homogenates from mice fed high fat diet. Candidate bioactive peptides were selected on the basis of species conservation, high expression/biosynthesis in EECs and evidence of regulated secretion*in vitro*. Candidate novel gut-derived peptides were chronically administered to mice to assess effects on food intake and glucose tolerance.

**Results:**

A large number of peptide fragments were identified from human and mouse, including known full-length gut hormones and enzymatic degradation products. EEC-specific peptides were largely from vesicular proteins, particularly prohormones, granins and processing enzymes, of which several exhibited regulated secretion *in vitro*. No regulated peptides were identified from previously unknown genes. High fat feeding particularly affected the distal colon, resulting in reduced peptide levels from GCG, PYY and INSL5. Of the two candidate novel peptides tested *in vivo*, a peptide from Chromogranin A (ChgA 435−462a) had no measurable effect, but a progastrin-derived peptide (Gast p59−79), modestly improved glucose tolerance in lean mice.

**Conclusion:**

LC–MS/MS peptidomic analysis of murine EECs and human GI tissue identified the spectrum of peptides produced by EECs, including a potential novel gut hormone, Gast p59−79, with minor effects on glucose tolerance.

## Highlights

• A peptidomic analysis of the human and mouse gastrointestinal tract was performed.
• High fat feeding reduced the content of GCG, PYY and INSL5 in the distal colon.
• Several interesting novel peptides were synthesised for *in vivo* characterisation.
• A novel product of progastrin modestly improved glucose tolerance in lean mice.

## 1 Introduction

Enteroendocrine cells (EECs) reside in the gastrointestinal (GI) epithelium and generate hormonal signals related to the rate of nutrient absorption from the GI tract [1]. There are at least 15 known peptide gut hormones with a variety of local and remote physiological effects. The ability of gut hormones to control glucose homeostasis and satiety has led to the development of long-acting glucagon-like peptide-1 (GLP-1) receptor agonists, which are now widely used in clinical practice to treat diabetes and obesity. A number of additional GLP-1 based peptides are in the pipeline, which combine the physiological activity of two or more endogenous peptides into a single synthetic pharmaceutical [[2], [3], [4], [5]]. The discovery of novel gut-derived peptide hormones with metabolic activity could thus lead to the development of new therapeutics to treat metabolic and gastrointestinal diseases.

Classical gut hormones such as cholecystokinin (CCK) and GLP-1 are cleaved from longer prohormones by prohormone convertases 1/3 and 2 (predominantly PC1/3 in the intestine) [6], which recognise mono- and dibasic cleavage sites. Proglucagon contains 3 known bioactive peptide sequences, and is cleaved to release GLP-1, GLP-2 and oxyntomodulin in the gut, or glucagon in pancreatic α-cells [7]. Proenkephalin [8,9] and proopiomelanocortin [10] also contain several distinct peptide sequences within a single prohormone, as identified in studies on the CNS. Whilst prohormone cleavage and processing are well characterised for the known bioactive gut hormones, the processing of other regions of these prohormones is poorly annotated, raising the possibility that they could contain previously unidentified bioactive peptides.

Many studies have claimed that active peptides are produced from chromogranin (Chg) A [11], arising because their vesicular location exposes them to processing enzymes. The wider granin family consists of secretory granule proteins believed to help stabilise vesicular structure, and includes ChgA, secretogranin (Scg) 1, Scg2 (or ChgB), Scg3, Scg4, Scg5 (or 7B2), Scg6, Scg7 (or VGF) and proSAAS [12]. They are believed to bind solutes such as catecholamines and Ca^2+^, reducing osmotic stress exerted on intracellular structures and preventing diffusion back into the cytosol [13]. With a high frequency of mono- and dibasic cleavage points in their sequences, these proteins are also processed by PC1/3 and PC2 to produce numerous peptides, some of which may exert physiological actions distinct from the intact protein [[14], [15], [16]]. Although many studies have identified candidate bioactive fragments of granin proteins, little work has been performed to characterise the breadth of peptide products produced from the wider group of vesicular proteins.

Many different approaches can be taken to identifying novel peptides. Corbière et al. outline 6 strategies used over the last 70 years to identify neuropeptides [17]. Peptidomic-based approaches have not been extensively used until recently, due to the specialist instrumentation required. Several studies have analysed the peptidome of pancreatic islets [18,19], hypothalamus [20,21], and EECs [22,23], but a comprehensive profile of peptides produced from prohormones, granins and processing enzymes is lacking. In this study, we aimed to identify peptides arising from precursor proteins highly expressed in EECs according to matching transcriptomic data. We did not identify peptides derived from previously unknown precursor proteins, but characterised the processing of prohormones, granins and PCs in EECs, and tested candidate peptides for bioactivity i*n vitro* and *in vivo*. We identified a peptide derived from progastrin (referred to as Gast p59−79) with modest effects on glucose tolerance in lean mice.

## 2 Materials and methods

### 2.1 Animals

All procedures were carried out with prior approval of the University of Cambridge and AstraZeneca Animal Welfare and Ethical Review Board and followed the regulations set out in the Animals (Scientific Procedures) Act 1986. All mice used were on a C57BL/6 N J background, unless indicated otherwise, and were sacrificed by a schedule 1 method. Animal work to characterise the peptidomics of enteroendocrine cells and the subcutaneous pharmacokinetics (PK) study was performed under project licences 70/7824 and PE50F6065. The intravenous pharmacokinetics studies were carried out under project licence P8A7322E2. *In vivo* efficacy studies on Gast p59−79 and ChgA 435−462a were carried out using either project licence P0C83A8BD or PF344F0A0.

### 2.2 Mouse EEC peptidomics

#### 2.2.1 Tissue digestion and staining

Male and female NeuroD1-Cre/EYFP [24] (mixed background, 3–10 generations backcrossed with C57BL/6) were culled by cervical dislocation. The GI tract was removed, flushed and divided into 5 regions; the stomach, proximal small intestines (SI) (first 10 cm), mid-SI (middle 10 cm), distal SI (last 10 cm) and large intestines. The muscle layer was removed from the small and large intestines. The stomach was digested in 1.3 mg/mL of Pronase E (Fisher Scientific, Waltham, MA, USA) in PBS at ambient temperature. The small and large intestines were digested by 5 × 10 min incubations in PBS containing 1 mM DTT (Sigma-Aldrich, St. Louis, MO, USA) and EDTA (Sigma-Aldrich) (5 mM EDTA for small intestinal tissue, 15 mM for large intestinal tissue). After digestion of all tissues, single cells were obtained by digesting with 0.25 μg/mL trypsin and EDTA (Life Technologies, Carlsbad, CA, USA) in Hank’s Buffered Saline Solution (HBSS) (Sigma-Aldrich), centrifuging (500 g, 5 min, 4⁰C), re-suspending in HBSS (with 0.1 % BSA (w/v) and 10 μM Y-27632 (Tocris Bioscience, Bristol, UK) then filtering with a 100 μm filter then a 50 μm filter. The cell suspensions were stained with 2 μg/mL DAPI (Sigma) and 5 μM Draq5 (BD Bioscience, Oxford, UK) to identify live cells when performing fluorescence activated cell sorting (FACS).

#### 2.2.2 FACS and sample preparation

FACS was carried out on single cell suspensions using a BD FACSJazz at the Cambridge NIHR BRC Cell Phenotyping Hub. Single cells were gated using forward scatter-height (FSC-H), side scatter-height and side scatter-width. Live cells were identified by gating for DAPI-negative yet Draq5-positive cells. Cells were further sorted based on their fluorescence in the YFP channel. YFP positive cells (EECs) were sorted into Protein LoBind Eppendorf tubes with 800 μL 80 % (v/v) acetonitrile (ACN) (Fisher Scientific) and centrifuged (12 000 g, 5 min, 4⁰C). The aqueous phase was collected and dried overnight in a centrifugal vacuum concentrator (Eppendorf, Stevenage, UK) and stored at -70⁰C.

#### 2.2.3 HFD peptidomics study

Male mice were fed either a high-fat diet (HFD) (60 % kcal fat, Research Diets (New Brunswick, NJ, USA), D12492) or a standard laboratory chow diet for 13 weeks. At the end of the 13 weeks, fasting blood glucose levels were measured following a 6 h fast. Mice were culled and the GI tracts dissected out. Pieces (∼30 mg) of mouse GI mucosa were flushed with PBS and homogenised in 250 μL 6 M guanidine hydrochloride (GuHCl) (Sigma-Aldrich) with Lyzing MatrixD (MP biomedicals) beads using a FastPrep-24 homogeniser. 4 cycles of 40 s shaking at 6 m/s was used to homogenise tissue. Proteins in samples were precipitated by addition of 80 % ACN and centrifuged (12 000 *g*, 5 min, 4⁰C). The aqueous phase was removed and dried overnight in a centrifugal evaporator.

#### 2.2.4 Sample preparation

On the day of analysis, dried samples were re-suspended in 0.1 % v/v formic acid (FA) (Sigma-Aldrich) in water and subjected to solid phase extraction (SPE) using a Waters HLB PRiME μElution SPE plate (Waters, Milford, MA), reduced and alkylated as described in Kay et al. [25].

### 2.3 Human tissue peptidomics

#### 2.3.1 Ethics

A local ethics review committee (09/H0308/24) approved human studies where tissue was collected from patients undergoing surgical resection by the Human Research Tissue Bank at Addenbrooke’s Hospital, Cambridge, UK. Informed consent from all donors was obtained prior to tissue collection and donor identities were kept anonymous. Tissue was obtained from the following regions of the human GI tract: stomach, duodenum, jejunum, ileum, ascending colon, sigmoid colon and rectum.

#### 2.3.2 Tissue homogenisation and sample preparation

Human tissue samples were acquired from 30 donors varying in age and gender and from all regions of the GI tract. Pieces (∼30 mg) of human GI mucosa were homogenised in 250 μL 6 M GuHCl (Sigma-Aldrich) with Lyzing MatrixD (MP biomedicals) beads using a FastPrep-24 homogeniser. 4 cycles of 40 s shaking at 6 m/s was used to homogenise tissue. Proteins in samples were precipitated by addition of 80 % ACN and centrifuged (12 000 *g*, 5 min, 4⁰C). Supernatants were removed and stored at -70⁰C till extraction. On the day of analysis, samples reconstituted in 0.1 % v/v FA in water, extracted using SPE, reduced and alkylated as described previously [25].

### 2.4 Primary mouse GI crypt secretion peptidomics

#### 2.4.1 Culture preparation

Primary murine mixed GI culture were generated as previously described [26,27]. Briefly, the small and large intestines were flushed with PBS and the stomach was inverted to remove contents. All GI tissue were minced and digested with collagenase XI (Stomach: 0.35 mg/mL, small intestines: 0.3 mg/mL, large intestines: 0.4 mg/mL) for 50 min at 37⁰C. Every 10−15 min fractions were collected and fresh DMEM with collagenase XI was added. Cells in each fraction were washed in DMEM (with 10 % FBS, 1% penicillin & streptomycin, 1% l-glutamine and 10 μM Y-27632), filtered using a 70 μm filter, pelleted and resuspended in DMEM. The crypt preparation was distributed evenly between 4 wells of a 12-well plate - which had been pre-coated with Matrigel (Corning) - and placed in a humidified incubator at 37⁰C.

### 2.5 GI crypt cell secretion assay

After culturing cells for 16−24 h, cells were washed twice with HEPES-buffered saline (see Kay et al. 2017 [25] for composition) and incubated at 37⁰C with the same HEPES-buffered saline for 1 h. The basal supernatant from each well of each tissue type was pooled into a Protein LoBind tube, cleared of debris by centrifuging at 2000 g for 5 min at 4⁰C and stored at -70⁰C. Immediately after removal of the basal supernatant, fresh HEPES-buffered saline with forskolin (Sigma-Aldrich) (10 μM), IBMX (Sigma-Aldrich) (10 μM) and glucose (10 mM) was added to each well. After a 1 h incubation at 37⁰C the stimulated supernatants from each well of each tissue type was removed, pooled and treated in the same way to the basal supernatant. On the day of analysis, supernatants were defrosted, spiked with 1% v/v FA in water to reach a final FA percentage of 0.1 % in the sample, extracted using SPE, reduced and alkylated.

### 2.6 Nano flow-rate mass spectrometry analysis and peptide identification

To achieve the greatest sensitivity and coverage, lysed murine EECs, supernatants from murine GI crypts and human GI tissue homogenates were analysed using a Q Exactive Plus Orbitrap mass spectrometer coupled to a Thermo Fisher Ultimate 3000 nano liquid chromatography system (Thermo Fisher Scientific) as previously described [25] except that the sample run time was 130 min. PEAKS v8.0 (Waterloo, Ontario, Canada) was used to identify peptides up to 65 amino acids in length against the human and mouse Swiss-Prot databases (downloaded on 26^th^ October 2017). In order to identify potential new translated sequences based on unannotated genes or splice variants, custom protein databases were generated based on the transcriptomics data from sorted mouse EECs (GSE114913, [28]). Reads from bamfiles were assembled using the mouse genome GRCm38.88 using Stringtie v1.3.1 [29] or Cufflink v2.2.1 [30]. GTF files were then converted into FASTA sequences using gffread v0.11.7 [31]. New sequences were identified by comparing peptides from the search on our custom database to the ones obtained using Swiss-Prot. As samples were reduced and alkylated, a fixed cysteine carbamidomethylation modification was used to search the data as well as variable N-terminal pyroglutamate, N-terminal acetylation and C-terminal amidation modifications. A subsequent PEAKS post-translational modification search was performed to identify up to 313 post-translation modification which may be present on peptides such as phosphorylation and octanoylation. Peptides matched were then plotted against their prepropeptide of origin using R v3.3.2. Peptides were quantified by integrating the area under the curve for selected *m/z* ranges and expressed as peak area. For peptides in alignment plots, quantification was performed by PEAKS.

### 2.7 Peptide synthesis and characterisation

Peptides were prepared as C-terminal carboxamides on NOVASYN® TGR resin and as C-terminal carboxylic acids on NOVASYN® TGA resin by standard Fmoc/tBu chemistry using HCTU/DIPEA in DMF on an automated peptide synthesizer (Prelude, Gyros Protein Technologies, USA). After deprotection and cleavage from the resin with a cocktail of TFA (95 % v/v), TIPS (2.5 % v/v), water (2.5 % v/v) for 3 h at RT, the peptides were precipitated in diethyl ether. The crude peptides were purified by reverse-phase HPLC (Varian) using a C8 column (Agilent Polaris, 21.2 × 250 mm, 5 μm) and a linear gradient elution of 5–50% acetonitrile containing 0.1 % TFA (v/v). To verify molecular masses against calculated theoretical values, purified peptides were characterized by single quadrupolar mass spectrometry using a Waters Mass Lynx 3100 platform. Positive electrospray ionisation (ESI) was used as the source. Analytes were loaded onto a C18 column (Waters X-Bridge, 4.6 × 100 mm, 3 μm), eluted using a linear gradient elution of 10–90 % acetonitrile with 0.1 % TFA over 10 min at 1.5 mL/min at RT and they were detected by both UV absorption at 210 nm and ionization using a Waters 3100 mass detector. Analytical RP-HPLC spectra were also recorded on an Agilent 1260 Infinity system using a linear gradient elution of 10–90 % acetonitrile with 0.1 % TFA over 15 min at 40 °C.

### 2.8 Pharmacokinetics studies

#### 2.8.1 Protocol

Male and female mice aged between 12–18 weeks old were used for these studies. Gast p59−79, ChgA 435−462a and Sst 25−36 were administered intravenously (i.v.) at 1 mg/kg and subcutaneously (s.c.) at various doses (see Fig. 7A, B and C for details on specific doses for each peptide). Blood samples were collected using EDTA coated microvette tubes (Starsedt, Leicester, UK) from the tail vein at various time points over 90 min. Blood was centrifuged (2000 *g*, 10 min, 4⁰C) and the plasma separated. A 0.1 M sodium citrate (Sigma-Aldrich) buffer (adjusted to pH 3 with citric acid (Sigma-Aldrich)) was added to the plasma at a dilution of 1 in 10 to prevent degradation of Gast p59−79 and Sst 25−36. Plasma was stored -70⁰C till extraction.

#### 2.8.2 Sample preparation

On the day of analysis, plasma samples were thawed, spiked with a stable isotope labelled version of Gast p59−79, ChgA 435−462a or Sst 25−36, and crashed with 200 μL 80 % ACN. After centrifuging, supernatants were removed and dried under gaseous nitrogen at 40⁰C using a SPE Dry evaporator system (Biotage, Upsalla, Sweden). SPE was performed as described previously [25].

#### 2.8.3 High flow rate mass spectrometry analysis

A Xevo TQ-XS triple quadrupole mass spectrometer coupled to H-class Aquity UPLC system enabled high throughput and targeted sample analysis. The column used was a Waters HSS T3 2.1 × 50 mm column at 60 °C and flowing at 350 μL/min. LC–MS/MS analysis was performed using positive ESI with a spray voltage of 3 kV, desolvation temperature 600°C, gas flow rate 1000 L/h and cone voltage of 40 V. Solvent A was 0.1 % FA in water (v/v) and Solvent B was 0.1 % FA in ACN (v/v). Each sample (40 μL per sample) was injected onto the column for 30 s with starting conditions set at 90 % A and 10 % B before the % B was increased to 50 % over the course of 3.5 min. The column was flushed with 90 % B for 1.5 min before initial conditions were restored. Total run time per sample was 5.5 min. Table 1 details the specifications used to quantify Gast p59−79, ChgA 435−462a, Sst 25−36 and their stable isotope labelled versions on a Xevo TQ-XS mass spectrometer.

**Table 1:** Specifications used to quantify peptides using the Xevo TQ-XS mass spectrometer. The precursor ions for each peptide are multiply charged and therefore can yield product ions with larger *m/z* values than the precursor ion as is the case for ChgA 435-462a, Sst 25-36 and their internal standards. IS: internal standard.

Peptide	Gast p59−79	Gast p59−79 IS	ChgA 435−462a	ChgA 435−462a IS	Sst 25−36	Sst 25−36 IS
Collision energy	18	18	18	18	18	18
Precursor (m/z)	477.7	479.2	635.9	638.9	476.6	478.9
Product (m/z)	470.5	471.9	721.4	725.8	529.3	532.73
Dwell time (ms)	0.04	0.04	0.04	0.04	0.04	0.04

### 2.9 *In vitro* osmotic pump assessment

The release of Gast p59−79 by 2004 model Alzet (Cupertino, CA, USA) osmotic pumps was assessed prior to performing *in vivo* efficacy studies. Gast p59−79 (4 mg/mL) was filled into 2 osmotic pumps. In 1 pump, 0.1 % (w/v) BSA had been dissolved in the peptide vehicle while the other did not have BSA. Each pump was placed in a separate Protein LoBind Eppendorf filled with PBS at 37 °C. Once every 4 days, pumps were transferred to a fresh Eppendorf and the old one stored at −70 °C. Control samples were generated by spiking in the estimated release volume from a 2004 Alzet pump over 4 days of 4 mg/mL Gast p59−79 and then incubating at 37 °C for 4, 8, 12, 16 or 20 days. An empty osmotic pump was also placed in the control tubes to account for peptide binding to the pump. Samples were then extracted by SPE and analysed as described in Section 2.8.3.

### 2.10 *In vivo* efficacy studies

Three studies were performed to assess the *in vivo* efficacy of the ChgA 435−462a and Gast p59−79 (see Figure legends for specifics of each study). Peptides were administered by osmotic pumps implanted subcutaneously under isoflurane anaesthesia. Food intake was assessed using BioDaq (Research Diets) automated food intake cages. Intraperitoneal (IP) glucose tolerance tests (GTT) were performed by administering a 2 g/kg challenge of glucose and measuring blood glucose levels from the tail vein whereas oral GTT (OGTT) were performed using 1 g/kg glucose challenge. Insulin tolerance tests (ITT) were performed by administering a 0.75 U/kg dose of insulin IP to the mice and assessing blood glucose levels at 15, 30, 45, 60, 90 and 120 min post-insulin challenge.

#### 2.10.1 Post-mortem assessment of insulin sensitivity

At the end of the 3^rd^ osmotic pump study, mice were given a 0.75 U/kg insulin challenge and culled after 15 min. The liver, white adipose tissue (WAT) and skeletal muscle were dissected out and snap frozen in liquid nitrogen and stored at −70 °C until analysis.

50−100 mg pieces of WAT, liver and skeletal muscle were homogenised in MSD (Rockville, MD, USA) lysis buffer with various protease inhibitors supplied by MSD and Qiagen metal bead. Homogenised tissue was allowed to rest at 4⁰C for 2 h to allow protein solubilisation and then centrifuged (10 000 *g*, 10 min, 4⁰C). Supernatants were aspirated and stored at -70⁰C till analysis. Protein content of all homogenised tissue samples was assessed using a Pierce BCA protein assay kit (Thermo Fisher Scientific) according to the manufacturer’s instructions.

Tissue homogenates were diluted in MSD lysis buffer to the same protein content. Total and phosphorylated-AKT (pAKT) were quantified using respective kits from MSD (total-AKT kit catalogue number: K150MOD-1, pAKT (Ser473) kit catalogue number: K151CAD-1) according to the manufacturer’s instructions.

### 2.11 Islet secretion studies

Mice were culled by cervical dislocation and the pancreas inflated by random injection of a 0.75 mg/mL collagenase V (Sigma-Aldrich) solution in HBSS before removal of the pancreas. The pancreas was incubated at 37⁰C for 12 min to liberate islets. The digested tissue was washed in HBSS with 0.1 % BSA before islets were hand-picked under a light microscope. Islets were rested for 1 h by incubating at 37⁰C in Krebs’s ringer buffer (KRB, 129 mM NaCl, 2 mM NaHCO_3_, 4.8 mM KCl, 1.2 mM KH_2_PO_4_, 2.5 mM CaCl_2_, 1.2 mM MgSO_4_, 10 mM HEPES and adjusted to pH 7.4 with 1 M NaOH.) with 0.1 % BSA and 5.5 mM glucose. Ten size matched islets were then transferred to a 2 mL protein LoBind Eppendorf tubes for 45 min at 37⁰C with KRB + 0.001% BSA + one of the following: 1 mM glucose, 16.7 mM glucose, 1 mM glucose + 100 nM Gast p59−79 or 16.7 mM glucose + 100 nM Gast p59−79. At the end of the incubation the supernatant was removed and snap frozen. On the day of analysis all supernatants were defrosted, acidified with 30 μL of 1% FA (aq) (v/v) and extracted with SPE.

Peptides were quantified on as in section 2.8.3. Table 2 details the peptides quantified as well as their product ions, precursor ions, collision energies and dwell times.

**Table 2:** Specifications used to quantify peptides in islet supernatants using the Xevo TQ-XS mass spectrometer. The precursor ions for each peptide are multiply charged and therefore can yield product ions with larger *m/z* values than the precursor ion as is the case for SST-14 and glucagon. SST-14: somatostatin-14.

	Insulin-1	SST-14	Glucagon
Collision energy	40	15	27
Precursor (m/z)	967.8	546.6	871.5
Product (m/z)	331.2	726.3	1040.2
Dwell time (ms)	0.025	0.025	0.025

### 2.12 Data analysis

Raw data files obtained on the Xevo TQ-XS triple quadrupole mass spectrometer were analysed using TargetLynx XS v4.2 software (Waters). Peptides were quantified by integrating the corresponding peaks and normalised to an internal standard if available. For pharmacokinetics studies, the concentration of peptide was estimated by comparing against a calibration line. SAAM II v2.2.1 (The Epsilon Group) with a Rosenbrock integrator and no maximum number of iterations was used to create a model for Gast p59−79 distribution and elimination upon single dose administration to mice.

## 3 Results

Our starting hypothesis was that novel bioactive intestinal peptides would exhibit some of the following features: a) Identifiable by peptide mass spectrometry in purified mouse EECs and human tissue biopsies, b) Expression at high levels in enteroendocrine cells at the mRNA level, c) Characteristic cleavage sites/patterns from longer precursor peptides, d) Relative sequence conservation between mouse and human, e) Altered biosynthesis following high fat feeding, f) Regulated secretion from primary intestinal cultures. EEC mRNA expression data from human and mouse have been reported previously [28,32].

### 3.1 Peptidomics of human and mouse prohormones and granin proteins

LC–MS/MS peptidomic analysis was performed on FACS-purified EECs from NeuroD1-Cre/YFP mice, and on human intestinal biopsies, from different regions of the GI tract. No enzymatic digestion of samples prior to LC–MS/MS analysis was performed so as to characterise the endogenous peptidome. PEAKS database searching of the combined data from all gut regions of 20 mice matched 5678 unique peptides originating from 933 unique proteins. The majority of identified peptides originated from prohormones, granins and processing enzymes, as well as cytoskeletal proteins and histones. None of the other peptides identified matched the criteria of being highly expressed in EECs at the mRNA level, so were not considered likely regulatory hormones. A similar analysis of homogenised human GI mucosa was performed on samples from 30 individual donors. PEAKS database search identified 13494 unique peptides from 2049 individual proteins. Compared with purified murine EECs, peptides originating from prohormones, granins and processing enzymes made up a smaller fraction of the peptidomic dataset, whereas peptides derived from cytoskeletal proteins, histones and proteins associated with the extracellular matrix dominated the results. This was not unexpected as EECs only comprise a minor cell population when working with whole tissue homogenates. Supplementary Tables S2 and S3 contain details of all peptides matched in FACS-purified murine EECs and human GI tissue homogenates respectively.

#### 3.1.1 Peptides from prohormones

**Fig. 1:**
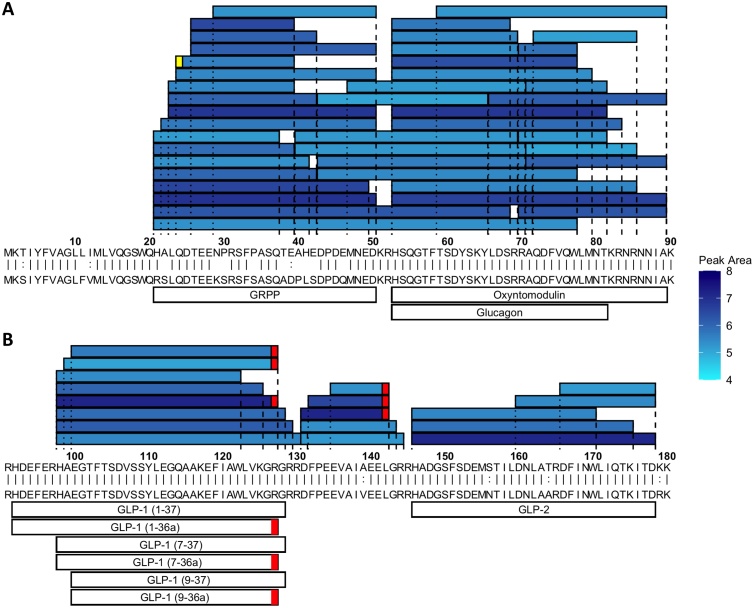
Peptides derived from preproglucagon with peak area >1e4 in NeuroD1 sorted cells from all regions of mouse the GI tract. (A) Peptides from positions 1-90. (B) Peptides from positions 91-180. In A and B murine prepropeptide (top sequence) is aligned to the human prepropeptide (bottom sequence) where ‘|’ represent identical residues and ‘:’ represent chemically similar amino acids. Shading in blue is a measure of the peptide’s peak area (log scale). Peptides described in literature are annotated on the bottom. Glicentin has not been annotated as it is too long to be matched by PEAKS. Red boxes indicate C-terminal amidations. Yellow boxes represent pyroglutamate residues. N.B glucagon was only detected in the stomach of murine EECs but at no other point along the GI tract.

To increase our understanding of the LC–MS/MS data and the peptide fragments identified, we first examined the peptides derived from preproglucagon (Figs. 1 and S1 – mouse and human). Reflecting the high abundance of this prohormone, we not only identified intact GLP-1, GLP-2 and oxyntomodulin, but also a number of partially digested fragments which were evident as “ladders”, differing by one or two amino acids at the N or C-terminus, reflecting proteolytic peptide degradation, likely during tissue/cell collection. The highest abundance GLP-1 peptide was GLP-1 (7−36amide), but several other GLP-1 peptides and fragments started with the same N-terminus, highlighting the dominance of this N-terminal cleavage site. We also detected GLP-1 forms starting at the 9-position (*e.g.* GLP-1 (9−36amide), known to result from dipeptidyl peptidase-dependent activity which inactivates this hormone), and in humans but not mice, 2 lower abundance peptides beginning at the 1-position. From the N-terminal half of proglucagon, we detected oxyntomodulin and glucagon but only in the murine stomach.

**Fig. 2:**
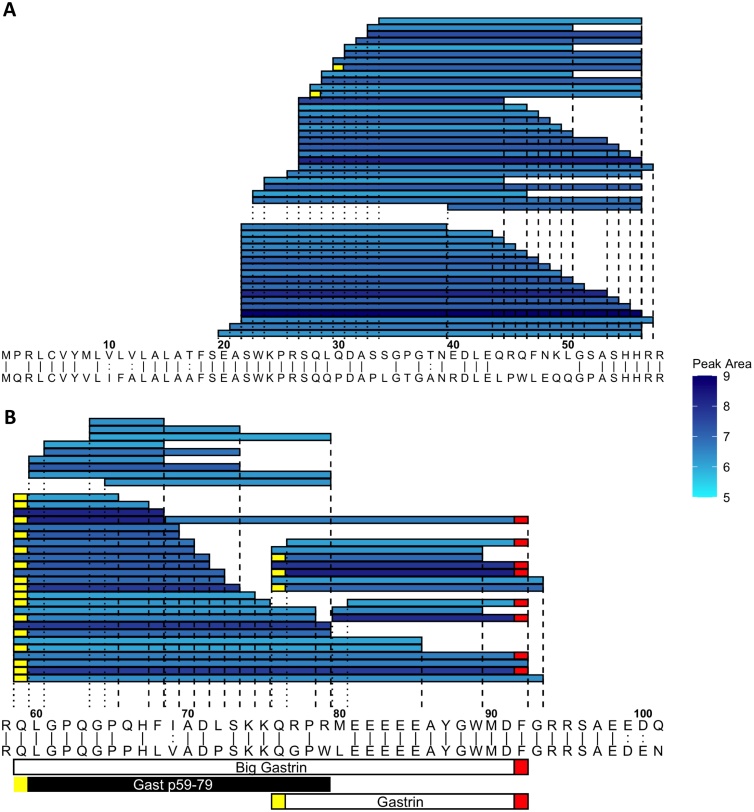
Peptides derived from preprogastrin with peak area >1e5 in NeuroD1 sorted cells from all regions of the mouse GI tract. (A) Peptides from positions 1-58. (B) Peptides from positions 59-101. In A and B murine prepropeptide (top sequence) is aligned to the human prepropeptide (bottom sequence) where ‘|’ represent identical residues and ‘:’ represent chemically similar amino acids. Shading in blue is a measure of each peptide’s peak area (log scale). Peptides described in literature are annotated on the bottom with peptide selected for synthesis in black (Gast p59-79). Red boxes indicate C-terminal amidations. Yellow boxes represent pyroglutamate residues.

We next examined peptide fragments from other prohormones with enriched mRNA expression in EECs. Very few peptides spanning signal peptide sequences were matched, indicating complete degradation of this sequence during processing. In progastrin we observed clear cleavage points flanking the sequence of big gastrin (a 34 amino acid peptide containing gastrin at the C-terminus) in mice (Fig. 2) and humans (Fig. S2). Whilst we also detected gastrin itself, a number of peptide fragments within big gastrin were not cleaved at the N-terminus of gastrin. From proCCK, PEAKS database searches were unable to match sulfated CCK forms, likely due to their low abundance caused by our methodological conditions and only by manually searching the raw data files were we able to detect sulfated CCK8. However, non-sulfated CCK8 and numerous other peptide fragments we matched from proCCK by PEAKS (Figs. S3, S4). A clear cleavage point towards the N-terminus of proCCK yielded a fragment Cckn p21−44 in both humans and mice. From proghrelin (Figs. S5, S6), we identified ghrelin itself (residues 24–51) in humans and mice, with the octanoylation and sometimes decanoylation occurring either at position S25 or S26 in mouse, but only at the previously described S26 position in human. We were unable to detect obestatin, previously described to originate from proghrelin [33]. Full length glucose-dependent insulinotropic polypeptide (GIP (1–42)) was identified in mouse and human (Figs. S7, S8), as well as the previously reported shorter version GIP (1–30) [34]. In human but not mouse, there was a small amount of GIP (3–42), suggesting dipeptidyl peptidase cleavage. We detected fully processed IAPP in murine EECs although a C terminal peptide seemed to be the most prevalent peptide derived from proIAPP (Fig. S9). No peptides derived from proIAPP were detected in human GI epithelium.

**Fig. 3:**
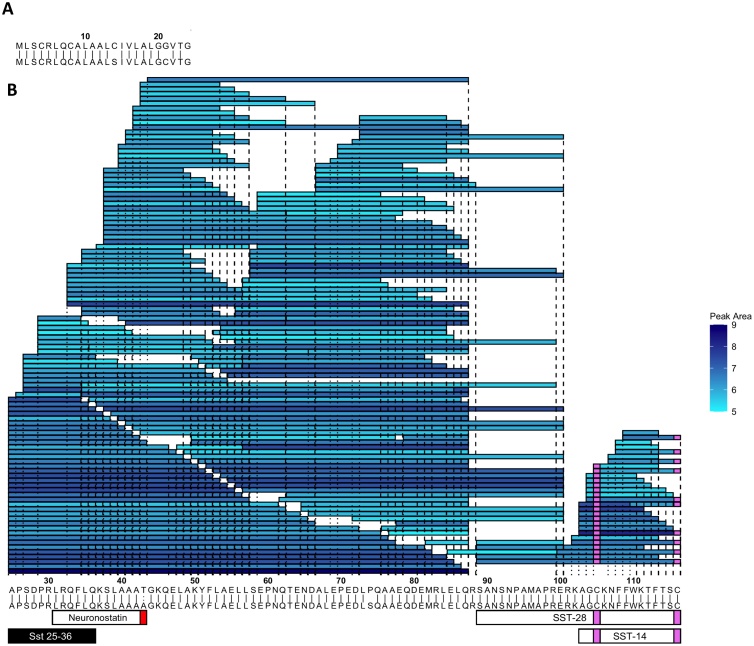
Peptides derived from preprosomatostatin with peak area >1e5 in NeuroD1 sorted cells from all regions of the mouse GI tract. (A) Peptides from positions 1-24. (B) Peptides from positions 25-116. In A and B murine prepropeptide (top sequence) is aligned to the human prepropeptide (bottom sequence) where ‘|’ represent identical residues and ‘:’ represent chemically similar amino acids. Shading in blue is a measure of each peptide’s peak area (log scale). Peptides described in literature are annotated on the bottom with peptide selected for synthesis in black (Sst 25-36). Red boxes indicate C-terminal amidations. Pink boxes represent carbamidomethylations.

Both neurotensin (Figs. S10, S11) and pancreatic polypeptide (PPY) (Fig. S12) were detected in humans and mice. PYY was identified in both full length (1–36) and shortened (3–36) forms and was predominantly amidated at the C-terminus (Figs. S13, S14) and occasionally phosphorylated at Prepro-PYY Serine-41. Secretin was the major product from prosecretin in both mice and humans (Figs. S15, S16). From prosomatostatin, we identified both SST-14 and SST-28, but no evidence of the previously annotated peptide neuronostatin (Figs. 3 & S17). Substance P and neurokinin A were identified from protachykinin in both human and mouse (Figs. S18 & S19). We identified only a small number of matches from PENK in mice, likely due to its low abundance (Fig. S20) but did not detect any PENK derived peptides in human GI tissue. We also looked for, but were unable to detect, xenin, a peptide previously described to originate from K-cells, but not arising from a classical vesicular protein [35]. Peptides matched from proINSL5, found in the colon, are not shown here as they have been published previously [25]. Peptides from splice variants of several preprohormones were detected and are displayed in Fig. S21.

#### 3.1.2 Peptides from granins and processing enzymes

A number of peptides were identified from the C-terminal region of ChgA, with evidence of several clear cleavage points (Fig. 4). We particularly noted the abundant fragment ChgA 435−462a, overlapping in sequence with the previously-described peptide serpinin, which has been shown to increase granule biogenesis and allosterically modulate β adrenoceptors [36,37]. Many peptides were also identified from other granins and processing enzymes (Figs. S23−33).

**Fig. 4:**
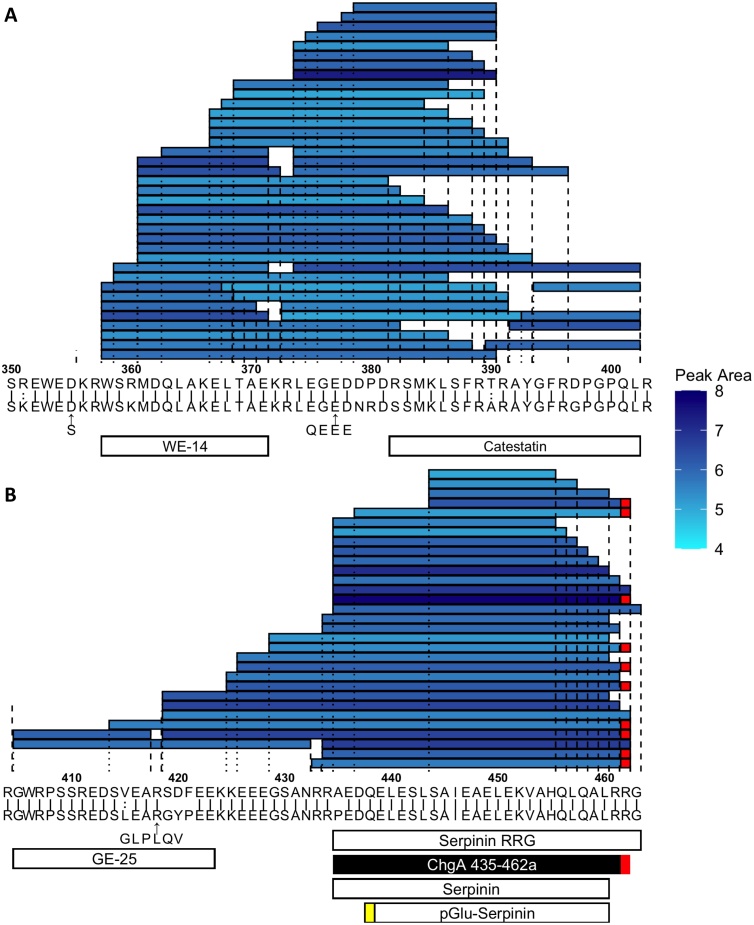
Peptides derived from the C-terminus of ChgA protein with peak area >1e4 in NeuroD1 sorted cells from all regions of the mouse GI tract. (A) Peptides from positions 350-403. (B) Peptides from positions 404-463. Murine prepropeptide (top sequence) is aligned to the human prepropeptide (bottom sequence) where ‘|’ represent identical residues and ‘:’ represent chemically similar amino acids. Shading in blue is a measure of each peptide’s peak area (log scale). Peptides described in Troger et al. 2017 [41] re annotated on the bottom. Parastatin is not annotated on the bottom as it is too long to be match by the PEAKS software. Peptide selected for synthesis in black (ChgA 435-462a). Red boxes indicate amidations. Yellow boxes represent pyroglutamate residues.

### 3.2 Effect of high fat diet on the intestinal peptidome

**Fig. 5:**
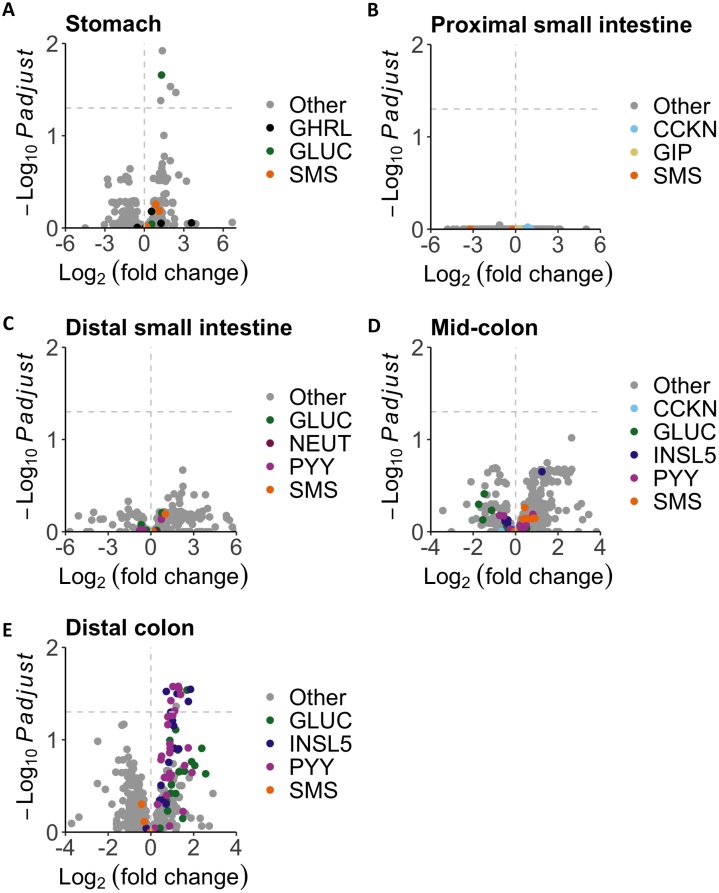
Peptidomic comparison between lean *vs* diet-induced obese (DIO) mice in (A) stomach, (B) proximal small intestine, (C) distal small intestine, (D) mid-colon and (E) distal colon. A positive log2 fold change value indicates a decrease in DIO mice. Peptides derived from EEC prohormones have been coloured in each plot. Peptides that weren't matched in at least 70 % of samples from one group were removed to reduce data complexity. As a result, some peptides such as those derived from prosecretin aren’t included in the plots.

To examine whether intestinal peptide processing is altered by dietary composition, we compared peptide profiles of intestinal samples from mice fed on chow or high fat diet (HFD), analysed by LC—MS/MS (Fig. 5). Significant differences in gut peptides between the groups were observed in the distal colon, but no major changes were detected in the small intestine. In the distal colon, HFD-fed mice exhibited lower levels of peptides from PYY, GCG and INSL5, consistent with our previous report of lower L-cell numbers in the colon but not small intestine of HFD-fed mice (Fig. 5E) [38]. We did not observe any appearance of alternative peptide fragments from these prohormones in the HFD group, suggesting the differences between groups occur at the level of propeptide biosynthesis not processing.

### 3.3 Analysis of peptide secretion

**Fig. 6:**
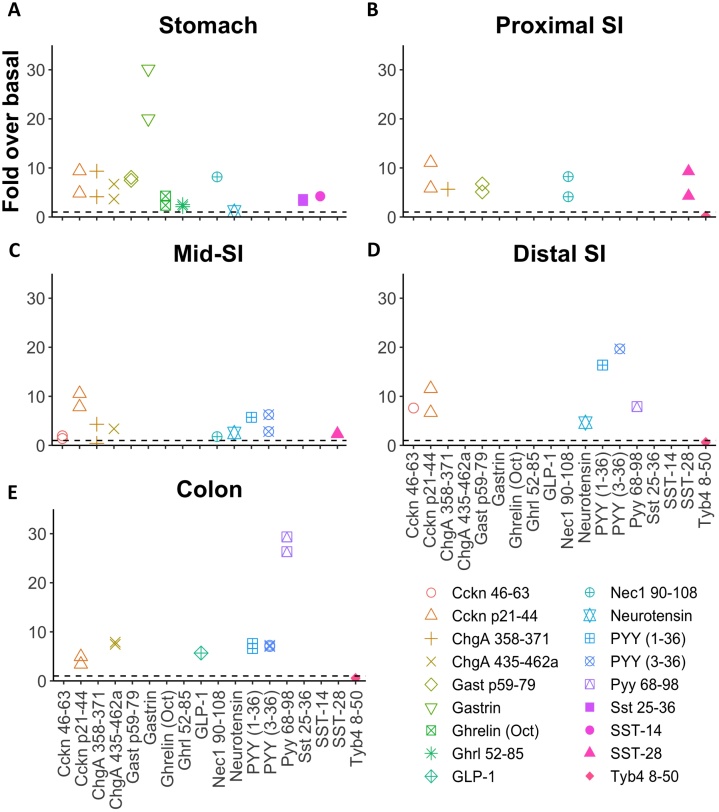
Peptide quantification in supernatants from primary mouse GI crypts. (A) Stomach, (B) proximal SI, (C) mid-SI, (D) distal SI, (E) colon. Fold over basal is calculated by normalising the peak area of the stimulated supernatant (10 μM forskolin, 100 μM IBMX, 10 mM glucose) to the peak area of the basal supernatant. Peptides such as GIP, insulin-like 5 and oxyntomodulin were not detected in basal supernatants and so no fold over basal value could be calculated. Tyb4 8-50 is an actin derived peptide. This peptide is not likely to be present in secretory vesicles of EECs and its release should not be stimulated by addition of forskolin, IBMX and glucose.

LC–MS/MS was performed on supernatants from primary intestinal epithelial cultures from different regions of the gut, treated with or without glucose, forskolin and IBMX, a cocktail that stimulates release of many gut hormones through elevation of cAMP. A number of peptides were detected from prohormones, granins and processing enzymes, and exhibited >2-fold enhanced release in glucose/forskolin/IBMX (Fig. 6). By contrast, peptides from the cytosolic protein thymosin beta 4 (Tyb4) [39] were detected but did not exhibit regulated secretion indicating that glucose, forskolin and IBMX does not induce non-specific release of all cellular peptides but only those associated with secretory granules. Most other EEC peptides we had identified in purified EECs and human tissue homogenates were not detectable in supernatants, likely because their concentrations were too low.

**Fig. 7:**
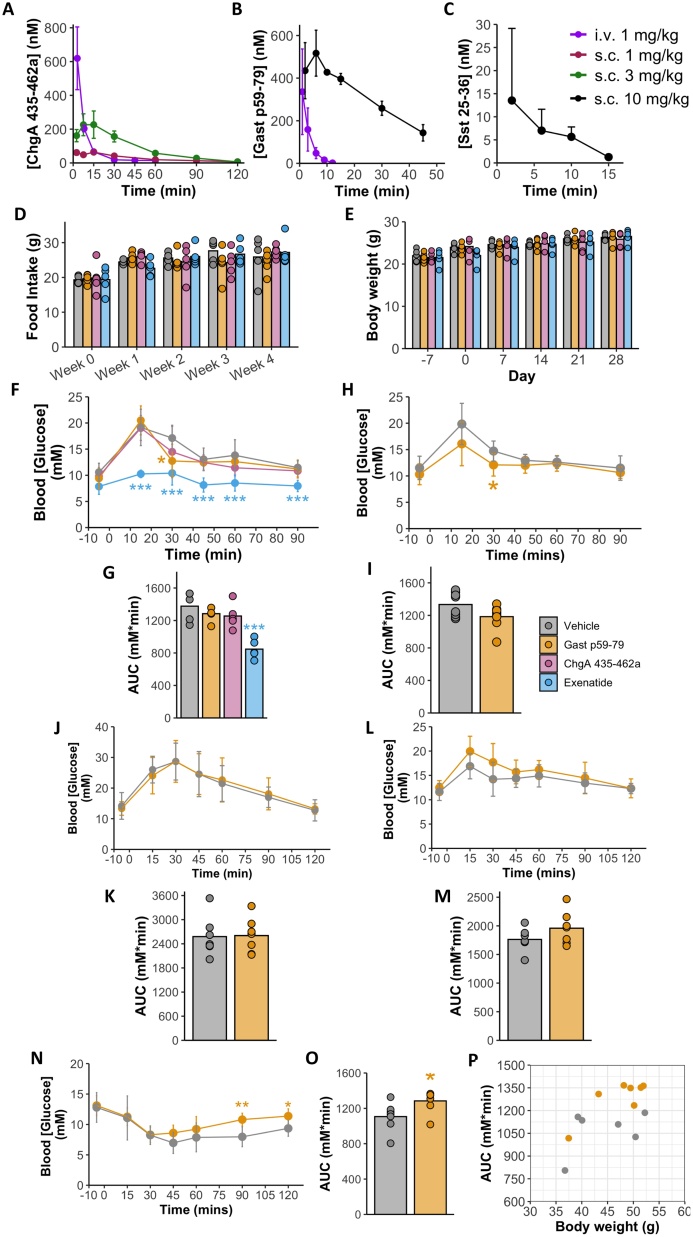
*In vivo* characterisation of novel peptides. (A-C) PK data of ChgA 435-462a, Gast p59-79, Sst 25-36. (D-G) Data from 1^st^ osmotic pump study where n = 5/group. 6-8 week old male mice were singly housed in BioDAQ food intake cages and infused with one of the following; ChgA 435-462a (0.3 mg/kg/day), Gast p59-79 (0.8 mg/kg/day), exenatide (0.03 mg/kg/day) or PBS (vehicle) *via* an osmotic pump for 28 days. Body weights (E) and food intake (D) were measured throughout the study. Week 0 and day -7 represent baseline data prior to treatment onset. Glucose tolerance was assessed every 7 days after surgery *via* an IP GTT using a 2 g/kg glucose challenge. Results from IP GTT on day 28 shown in (F, G). Statistical analysis performed using Kruskal-Wallis test with post-hoc Dunn tests. (H, I) Results from osmotic pump study 2 where n = 8/group. Mice singly housed and infused with either Gast p59-79 or vehicle for 7 days. 2 g/kg IP GTT performed on day 7 and results shown in H and I. Statistical analysis performed using T test. (J-P) Results from osmotic pump study 3 where n = 7/group. Mice singly housed and infused with either Gast p59-79 or vehicle for 7 days. 2 g/kg IP GTT performed on day 7 (J, K) and 1 g/kg OGTT performed on day 14 (L, M). 0.75 U/kg ITT performed on day 21 (N, O). Body weights *vs* AUC during ITT on day 21 (P). Error bars represent SD. *P < 0.05, **p < 0.01, ***p < 0.005.

### 3.4 Identification and characterisation of putative bioactive peptides

From the data presented above, we constructed a list of candidate peptides for analysis of potential bioactivity. This list was constructed by identifying peptides with ‘hormonal-like’ characteristics such as; being flanked by basic residues, highly abundant in human and mouse GI tissue, conserved with the equivalent human sequence and secreted from the GI epithelium in response to a stimulus. We also took into account similar peptides that previous literature had described as being bioactive but were not matched in our peptidomic data. Based on these criteria, three peptides, ChgA 435−462a, Gast p59−79 and Sst 25−36, were considered promising and selected for *in vivo* studies in mouse models. *In vitro*, all three peptides were stable in physiological buffer, and released in a controlled fashion from osmotic minipumps (data not shown). They were then tested for *in vivo* stability by bolus subcutaneous and i.v. injections in mice, with serial blood sampling and peptide measurement by LC–MS/MS (Fig. 7A–C & table S1). Sst 25−36 was undetectable in plasma after i.v. injection, and only detectable at low concentrations after the highest s.c. dose of 10 mg/kg, so was excluded from further studies. Gast p59−79 exhibited good bioavailability (table S1) but a short half-life (18.1 min) when administered subcutaneously. The bioavailability of ChgA 43−462a was calculated to be only ∼50 % (table S1) when administered s.c. but displayed a longer half-life than Gast p59−79 (36.0 min). The mass delivery rates required to obtain steady state concentrations of 1 nM Gast p59−79 or ChgA 435−462a were estimated to be 0.77 and 0.29 mg/kg/day, respectively. These mass delivery rates were used in subsequent *in vivo* studies to assess the effect of these peptides on metabolic parameters.

#### 3.4.1 Effects of Gast p59−79 and ChgA 435−462a on metabolic parameters *in vivo*

Gast p59−79 and ChgA 435−462a were infused into mice for 28 days using osmotic pumps, with measurements of body weight, food intake and weekly assessment of IP glucose tolerance. PBS (vehicle) and exenatide (0.03 mg/kg/day) were infused into separate groups as negative and positive controls, respectively. No significant differences were seen in body weight or food intake of mice receiving Gast p59−79 or ChgA 435−462a compared with controls (Fig. 7D, E). Surprisingly, we were also not able to observe a significant change in body weight or food intake in the exenatide treated mice. However, exenatide improved glucose tolerance in all 4 IP GTTs, as expected (data only shown for week 4 IP GTT, Fig. 7F, G), whilst ChgA 435−462a had no measurable effect. Small but significant improvements in individual time point glucose levels were observed in mice receiving Gast p59−79, although this did not reach significance in the AUC analyses (Fig. 7F, G). The effect of Gast p59−79 on glucose tolerance was replicable in a second mouse cohort (Fig. 7H, I), suggesting that it modestly improves glucose tolerance.

In a third mouse cohort, we examined the effects of Gast p59−79 on insulin tolerance, oral glucose tolerance and insulin signalling in diet-induced obese mice (DIO). Inexplicably, a number of mice (including controls) lost weight in the week following pump implantation, confounding interpretation of the glucose tolerance data. In this obese group, however, the results did not suggest that Gast p59−79 affected glucose tolerance (Fig. 7J–M). In ITTs, blood glucose levels of the two treatment groups differed at 90 and 120 min post-insulin challenge, as also evident in the AUCs (p = 0.011) but interpretation of these results is complicated when taking into account the significant effect of body weight on AUC (p = 0.009) using a two-way ANOVA.

#### 3.4.2 Effects of Gast p59−79 on insulin sensitivity and secretion

**Fig. 8:**
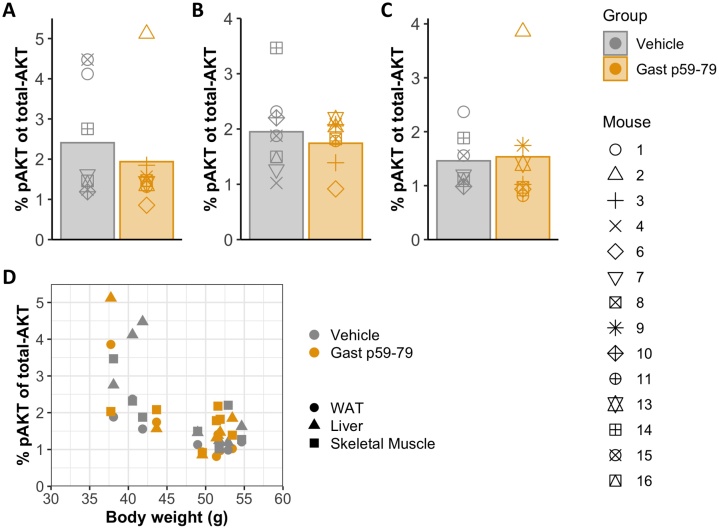
Phosphorylated-AKT (pAKT) expressed as a percentage of total AKT in liver (A), skeletal muscle (B) and white adipose tissue (WAT) (C) 15 min after a 0.75 U/kg insulin challenge. (D) Body weights *vs* pAKT as a percentage of total-AKT. T tests performed to analyse difference in % of pAKT between treatment groups in each tissue type. Additionally, a two-way ANOVA was used to investigate the effect of both treatment group and weight on % pAKT.

**Fig. 9:**
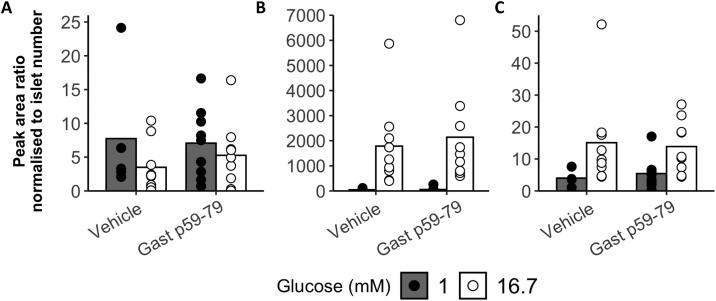
Effect of Gast p59-79 on glucagon (A), insulin-1 (B) and SST-14 (C) secretion from isolated mouse islets. Data represents n = 9 per group. Statistical analysis was performed using a Mann-Whitney U test. Data expressed as peak area normalised to islet number.

28 days after surgery, DIO mice were culled 15 min following a humulin challenge and tissue collected for assessment of insulin signalling by quantification of phosphorylated-AKT (pAKT) and total-AKT. No effect of treatment group on percentage of pAKT was found in any tissue (Fig. 8) although we did observe a significant effect of body weight on pAKT in all tissue types when using a two-way ANOVA (liver p = 0.0014, WAT p = 1.5e-4, skeletal muscle p = 0.0219, Fig. 8D). In WAT a significant interaction was found between weight and treatment group (p = 0.015), and no conclusions could be drawn about the individual effects of treatment group or weight on pAKT. To assess pancreatic hormone release, Gast p59−79 was applied to primary mouse islets *in vitro*, with quantification of insulin, glucagon and SST-14 in supernatants. No significant effect of Gast p59−79 was seen on glucagon, insulin-1 or SST-14 secretion at either 1 or 16.7 mM glucose (Fig. 9).

## 4 Discussion

In this study we characterised the processing of prohormones, granins and prohormone convertases in murine EECs and human GI tissue. All previously well-established gut hormones were detected in our data set although sulfated forms of CCK were present at low levels likely due to the fact that using positive ESI makes it difficult to detect peptides with few basic residues and with a sulfation [40]. Furthermore, peptides longer than 65 amino acids cannot be matched by the PEAKS software and so peptides such as glicentin would not have been identified. Neither obestatin nor neuronostatin were detected from proghrelin or prosomatostatin, respectively, and there was no evidence that either of these prohormones is cleaved at sites that could generate these peptides. It is possible that shortfalls in our methodology prevented the detection of neuronostatin or obestatin but this is unlikely as these peptides are shorter than 65 amino acids and both contain basic residues and thus should be positively charged in acidic conditions enabling good retention through our extraction process. Therefore, it is unclear as to whether either is actually produced in the human or mouse GI tract. Our approach did not identify any peptides from unknown precursors that satisfied the conditions of being EEC enriched, found in both mouse and humans, and exhibiting regulated secretion from primary intestinal cultures.

Bioactive granin-derived peptides have been described previously, and reported to act as paracrine or autocrine factors. The ability of peptides derived from granins or processing enzymes to act as specific hormonal signals between different tissues is limited because they are expressed in numerous secretory cell types which respond to different physiological states [11,41]. With the proviso that processing might not be identical between cell types, granin peptides could, for example, be released together with adrenaline from chromaffin cells in response to stress, with insulin from β cells in response to hyperglycaemia, from α-cells with glucagon in response to hypoglycaemia, or together with a range of gut peptides post-prandially.

Several studies have attempted to identify novel peptides using computer algorithms to predict conserved cleavage sites at mono- or dibasic residues at the C- or N-termini [[42], [43], [44]]. Applying this to the gut, one study generated a custom transcriptomic database of murine EECs by bulk RNA-seq, allowing prediction of peptides from previously undiscovered genes [42], but whilst this approach benefits from the application of high throughput analysis, the peptides predicted may only exist *in silico* and require validation by a technique such as MS/MS.

Peptidomic-driven approaches have been used previously for novel peptide discovery [18,20]. LC–MS/MS analyses of mouse and human intestinal biopsies and human organoid-derived EECs and supernatants have confirmed production of known hormones, but did not seek to identify unknown peptide species [23,25,28,32]. We have previously performed a similar study to compare the peptidome of human and mouse GI tissue, however this previous study focused on known hormones without providing much information on the processing of prohormones [28]. In the present study we sought to provide a comprehensive overview of the EEC peptidome of mice whilst focusing on the specific processing of prohormones, granins and processing enzymes and identifying novel and potentially active gut peptides. Identifying the exact sequence of physiological peptides by LC–MS/MS is complicated by the simultaneous identification of multiple peptide fragments generated by non-specific enzymatic degradation during sample preparation. Extensive peptide degradation was evident in supernatants from organoid cultures, collected after a 24 h incubation [23], which was reduced in the current study by incubating cells for shorter periods in simple saline buffer. Secher et al. addressed the issue of enzymatic peptide degradation in tissue samples post-sample collection by perfusing protease inhibitors as well as heat inactivation of enzymes in dissected rat hypothalamus [20], but peptide ladders generated by non-specific enzyme degradation were still observed. In that study, peptide ladders were removed by an algorithm that merged overlapping peptides into the longest peptide variant, an approach that reduces complexity of the data but loses information about potential peptide variants. Our method of processing tissue samples rapidly in either 80 % ACN or GuHCl was moderately successful in preventing peptide degradation in freshly isolated samples, but we still observed development of fragment ladders in purified EECs that had undergone the longer process of tissue dissociation and FACS sorting prior to GuHCl treatment. We avoided using protease inhibitors as a method of minimising peptide degradation as these protease inhibitors would interfere with peptide identification and shorten column life. Thus some *ex vivo* peptide degredation was inevitable. However the issue of peptide degradation producing fragment ladders can be somewhat addressed by filtering out peptides that have a signal intensity of <10 % of the most abundant peptide from the same propeptide, thereby reducing data complexity.

We also examined the effect of a HFD on the peptidome of murine GI tissue, and identified a specific reduction in L-cell related peptides in the distal colon. This is consistent with our previous finding that high fat feeding impaired L-cell numbers in the colon [38]. However, this study does not shed light on the mechanism by which a HFD may alter L-cell numbers and why the effects seem specific to the colon. It is possible that HFD alters the gut microbiota which in turn impacts on the number and function of L-cells as previously suggested from studies on germ-free mice [45].

From our peptidomic data, we highlighted three novel peptide hormone candidates for *in vivo* studies, two were derived from prohormones (Gast p59−79 and Sst 25−36) and the third from a granin protein (ChgA 435−462a). Sst 25−36 was excluded from further studies after pilot data suggested a very short half-life *in vivo*. Neither Gast p59−79 nor ChgA 435−462a had effects on body weight or food intake. Gast p59−79 modestly improved glucose tolerance in lean mice, and in DIO mice appeared to slow glucose normalisation following an ITT. However, we were unable to demonstrate direct effects on islet hormone secretion or insulin signalling quantified by pAKT in liver, WAT or skeletal muscle [46]. The effects seen in DIO mice should be treated with caution due to the confounding factor of weight differences in this study.

## 5 Conclusion

This LC–MS/MS peptidomic analysis aimed to identify new bioactive peptides from the GI tract. No new candidate hormones were identified from previously unknown genes, but we identified a variety of peptides generated by vesicular processing of prohormones, granins and processing enzymes. Of the candidates selected for further analysis, Gast p59−79 induced a modest improvement in glucose tolerance in lean mice but not in DIO mice. This comprehensive description of EEC peptide processing benefits from providing exact start and end points as well as post-translational modifications, which are difficult to distinguish with many antibody-based techniques. As we were only able to select a small proportion of candidates for functional evaluation, higher throughput methods will be required to determine whether the long list of identified peptides contains any with bioactivity, for example by screening against known GPCRs *in vitro* or *in silico*.

## Contribution statement

FMG, FR and LJ conceived the study with SGG, PL, FMG, FR, LJ, CA, DB, and RGK designing the experiments. SGG, PL, RGK and GPR performed the peptidomic experiments on FACS purified EECs and human GI tissue. SGG, VL and EO’F conducted the HFD mouse peptidomics study. EB synthesised the novel peptides. SGG, HP, HB, LS, SC, AM and DA-K designed and conducted the *in vivo* efficacy and PK studies on the 3 novel peptides. JH carried out the analysis of the PK data and modelling for *in vivo* administration. SGG carried out *in vitro* experiments to assess pAKT and islet secretions. SGG, FMG and FR wrote the manuscript which was reviewed and edited by the other authors. Intravenous PK, peptide synthesis and osmotic pump studies were performed at AstraZeneca (Cambridge, UK) whereas all other experiments were performed at the Institute of Metabolic Science.

## Funding

SGG is supported by a grant from the 10.13039/501100000268Biotechnology and Biological Sciences Research Council and 10.13039/100004325AstraZeneca. Research in the laboratories of FMG and FR is supported by 10.13039/501100000265Medical Research Council (MRC_MC_UU_12012/3) and 10.13039/100004440Wellcome Trust (106262/Z/14/Z and 106263/Z/14/Z). EO’F is supported by a grant from the Wellcome Trust. The MS instruments were funded by the MRC “Enhancing UK clinical research” grant (MR/M009041/1). Support for the core facilities at the Metabolic Research Laboratories was provided by the Medical Research Council (MRC_MC_UU_12012/5) and Wellcome Trust (100574/Z/12/Z).

## Data availability

The mass spectrometry proteomics data have been deposited to the ProteomeXchange Consortium *via* the PRIDE (see Perez-Riverol Y, Csordas A, Bai J, et al. 2020, PMID: 30395289) partner repository with the dataset identifiers PXD024050 (10.6019/PXD024050) and PXD009788 (10.6019/PXD009788). Transcriptomics data used to generate a custom proteome database of mouse EECs was deposited in the NCBI GEO repository (GSE114913).

For supplementary figures and supplementary tables S2 and S3 see accompanying documents.

## Declaration of Competing Interest

FMG is a consultant at Kallyope (New York, NY). FMG and FR receive support from AstraZeneca and Eli Lilly. All other authors declare no other conflicts of interest.

## References

[1] Gribble F.M., Reimann F. (2016). Enteroendocrine cells: chemosensors in the intestinal epithelium. Annu. Rev. Physiol..

[2] Pocai A., Carrington P.E., Adams J.R., Wright M., Eiermann G., Zhu L., Du X., Petrov A., Lassman M.E., Jiang G., Liu F., Miller C., Tota L.M., Zhou G., Zhang X., Sountis M.M., Santoprete A., Capito’ E., Chicchi G.G., Thornberry N., Bianchi E., Pessi A., Marsh D.J., SinhaRoy R. (2009). Glucagon-like peptide 1/glucagon receptor dual agonism reverses obesity in mice. Diabetes.

[3] Coskun T., Sloop K.W., Loghin C., Alsina-Fernandez J., Urva S., Bokvist K.B., Cui X., Briere D.A., Cabrera O., Roell W.C., Kuchibhotla U., Moyers J.S., Benson C.T., Gimeno R.E., D’Alessio D.A., Haupt A. (2018). LY3298176, a novel dual GIP and GLP-1 receptor agonist for the treatment of type 2 diabetes mellitus: From discovery to clinical proof of concept. Mol. Metab..

[4] Frias J.P., Nauck M.A., Van J., Kutner M.E., Cui X., Benson C., Urva S., Gimeno R.E., Milicevic Z., Robins D., Haupt A. (2018). Efficacy and safety of LY3298176, a novel dual GIP and GLP-1 receptor agonist, in patients with type 2 diabetes: a randomised, placebo-controlled and active comparator-controlled phase 2 trial. Lancet.

[5] Frias J.P., Nauck M.A., Van J., Benson C., Bray R., Cui X., Milicevic Z., Urva S., Haupt A., Robins D.A. (2020). Efficacy and tolerability of tirzepatide, a dual glucose-dependent insulinotropic peptide and glucagon-like peptide-1 receptor agonist in patients with type 2 diabetes: a 12-week, randomized, double-blind, placebo-controlled study to evaluate different dose-escalation regimens. Diabetes Obes. Metab..

[6] Gagnon J., Mayne J., Mbikay M., Woulfe J., Chrétien M. (2009). Expression of PCSK1 (PC1/3), PCSK2 (PC2) and PCSK3 (furin) in mouse small intestine. Regul. Pept..

[7] Holst J.J. (2007). The physiology of glucagon-like peptide 1. Physiol. Rev..

[8] Goumon Y., Strub J.M., Moniatte M., Nullans G., Poteur L., Hubert P., Van Dorsselaer A., Aunis D., Metz-Boutigue M.H. (1996). The C-terminal bisphosphorylated proenkephalin-A-(209-237)-peptide from adrenal medullary chromaffin granules possesses antibacterial activity. Eur. J. Biochem..

[9] Metz-Boutigue M.H., Goumon Y., Lugardon K., Strub J.M., Aunis D. (1998). Antibacterial peptides are present in chromaffin cell secretory granules. Cell. Mol. Neurobiol..

[10] Pritchard L.E., Turnbull A.V., White A. (2002). Pro-opiomelanocortin processing in the hypothalamus: impact on melanocortin signalling and obesity. J. Endocrinol..

[11] Helle K.B., Metz-Boutigue M.H., Cerra M.C., Angelone T. (2018). Chromogranins: from discovery to current times. Pflugers Arch..

[12] Machado J.D., Díaz-Vera J., Domínguez N., Alvarez C.M., Pardo M.R., Borges R. (2010). Chromogranins A and B as regulators of vesicle cargo and exocytosis. Cell. Mol. Neurobiol..

[13] Helle K.B., Reed R.K., Pihl K.E., Serck-Hanssen G. (1985). Osmotic properties of the chromogranins and relation to osmotic pressure in catecholamine storage granules. Acta Physiol. Scand..

[14] Doblinger A., Becker A., Seidah N.G., Laslop A. (2003). Proteolytic processing of chromogranin A by the prohormone convertase PC2. Regul. Pept..

[15] Eskeland N.L., Zhou A., Dinh T.Q., Wu H., Parmer R.J., Mains R.E., O’Connor D.T. (1996). Chromogranin A processing and secretion: specific role of endogenous and exogenous prohormone convertases in the regulated secretory pathway. J. Clin. Invest..

[16] Udupi V., Lee H.M., Kurosky A., Greeley G.H. (1999). Prohormone convertase-1 is essential for conversion of chromogranin A to pancreastatin. Regul. Pept..

[17] Corbière A., Vaudry H., Chan P., Walet-Balieu M.L., Lecroq T., Lefebvre A., Pineau C., Vaudry D. (2019). Strategies for the identification of bioactive neuropeptides in vertebrates. Front. Neurosci..

[18] Taylor S.W., Nikoulina S.E., Andon N.L., Lowe C. (2013). Peptidomic profiling of secreted products from pancreatic islet culture results in a higher yield of full-length peptide hormones than found using cell lysis procedures. J. Proteome Res..

[19] Stewart K.W., Phillips A.R., Whiting L., Jüllig M., Middleditch M.J., Cooper G.J. (2011). A simple and rapid method for identifying and semi-quantifying peptide hormones in isolated pancreatic islets by direct-tissue matrix-assisted laser desorption ionization time-of-flight mass spectrometry. Rapid Commun. Mass Spectrom..

[20] Secher A., Kelstrup C.D., Conde-Frieboes K.W., Pyke C., Raun K., Wulff B.S., Olsen J.V. (2016). Analytic framework for peptidomics applied to large-scale neuropeptide identification. Nat. Commun..

[21] Svensson M., Sköld K., Svenningsson P., Andren P.E. (2003). Peptidomics-based discovery of novel neuropeptides. J. Proteome Res..

[22] Egerod K.L., Engelstoft M.S., Grunddal K.V., Nøhr M.K., Secher A., Sakata I., Pedersen J., Windeløv J.A., Füchtbauer E.M., Olsen J., Sundler F., Christensen J.P., Wierup N., Olsen J.V., Holst J.J., Zigman J.M., Poulsen S.S., Schwartz T.W. (2012). A major lineage of enteroendocrine cells coexpress CCK, secretin, GIP, GLP-1, PYY, and neurotensin but not somatostatin. Endocrinology.

[23] Beumer J., Puschhof J., Bauzá-Martinez J., Martínez-Silgado A., Elmentaite R., James K.R., Ross A., Hendriks D., Artegiani B., Busslinger G.A., Ponsioen B., Andersson-Rolf A., Saftien A., Boot C., Kretzschmar K., Geurts M.H., Bar-Ephraim Y.E., Pleguezuelos-Manzano C., Post Y., Begthel H., van der Linden F., Lopez-Iglesias C., van de Wetering W.J., van der Linden R., Peters P.J., Heck A.J.R., Goedhart J., Snippert H., Zilbauer M., Teichmann S.A., Wu W., Clevers H. (2020). High-resolution mRNA and secretome atlas of human enteroendocrine cells. Cell.

[24] Li H.J., Kapoor A., Giel-Moloney M., Rindi G., Leiter A.B. (2012). Notch signaling differentially regulates the cell fate of early endocrine precursor cells and their maturing descendants in the mouse pancreas and intestine. Dev. Biol. (Basel).

[25] Kay R.G., Galvin S., Larraufie P., Reimann F., Gribble F.M. (2017). Liquid chromatography/mass spectrometry based detection and semi-quantitative analysis of INSL5 in human and murine tissues. Rapid Commun. Mass Spectrom..

[26] Habib A.M., Richards P., Rogers G.J., Reimann F., Gribble F.M. (2013). Co-localisation and secretion of glucagon-like peptide 1 and peptide YY from primary cultured human L cells. Diabetologia.

[27] Reimann F., Habib A.M., Tolhurst G., Parker H.E., Rogers G.J., Gribble F.M. (2008). Glucose sensing in L cells: a primary cell study. Cell Metab..

[28] Roberts G.P., Larraufie P., Richards P., Kay R.G., Galvin S.G., Miedzybrodzka E.L., Leiter A., Li H.J., Glass L.L., Ma M.K.L., Lam B., Yeo G.S.H., Scharfmann R., Chiarugi D., Hardwick R.H., Reimann F., Gribble F.M. (2019). Comparison of human and murine enteroendocrine cells by transcriptomic and peptidomic profiling. Diabetes.

[29] Pertea M., Kim D., Pertea G.M., Leek J.T., Salzberg S.L. (2016). Transcript-level expression analysis of RNA-seq experiments with HISAT, StringTie and Ballgown. Nat. Protoc..

[30] Trapnell C., Williams B.A., Pertea G., Mortazavi A., Kwan G., van Baren M.J., Salzberg S.L., Wold B.J., Pachter L. (2010). Transcript assembly and quantification by RNA-Seq reveals unannotated transcripts and isoform switching during cell differentiation. Nat. Biotechnol..

[31] Pertea G., Pertea M. (2020). GFF utilities: GffRead and GffCompare. F1000Res.

[32] Goldspink D.A., Lu V.B., Miedzybrodzka E.L., Smith C.A., Foreman R.E., Billing L.J., Kay R.G., Reimann F., Gribble F.M. (2020). Labeling and characterization of human GLP-1-secreting L-cells in primary ileal organoid culture. Cell Rep..

[33] Zhang J.V., Ren P.G., Avsian-Kretchmer O., Luo C.W., Rauch R., Klein C., Hsueh A.J. (2005). Obestatin, a peptide encoded by the ghrelin gene, opposes ghrelin’s effects on food intake. Science.

[34] Fujita Y., Yanagimachi T., Takeda Y., Honjo J., Takiyama Y., Abiko A., Makino Y., Haneda M. (2016). Alternative form of glucose-dependent insulinotropic polypepide and its physiology. J. Diabetes Investig..

[35] Anlauf M., Weihe E., Hartschuh W., Hamscher G., Feurle G.E. (2000). Localization of xenin-immunoreactive cells in the duodenal mucosa of humans and various mammals. J. Histochem. Cytochem..

[36] Koshimizu H., Cawley N.X., Kim T., Yergey A.L., Loh Y.P. (2011). Serpinin: a novel chromogranin A-derived, secreted peptide up-regulates protease nexin-1 expression and granule biogenesis in endocrine cells. Mol. Endocrinol..

[37] Tota B., Gentile S., Pasqua T., Bassino E., Koshimizu H., Cawley N.X., Cerra M.C., Loh Y.P., Angelone T. (2012). The novel chromogranin A-derived serpinin and pyroglutaminated serpinin peptides are positive cardiac β-adrenergic-like inotropes. FASEB J..

[38] Richards P., Pais R., Habib A.M., Brighton C.A., Yeo G.S., Reimann F., Gribble F.M. (2015). High fat diet impairs the function of glucagon-like peptide-1 producing L-cells. Peptides.

[39] Erickson-Viitanen S., Ruggieri S., Natalini P., Horecker B.L. (1983). Distribution of thymosin beta 4 in vertebrate classes. Arch. Biochem. Biophys..

[40] Nemeth-Cawley J.F., Karnik S., Rouse J.C. (2001). Analysis of sulfated peptides using positive electrospray ionization tandem mass spectrometry. J. Mass Spectrom..

[41] Troger J., Theurl M., Kirchmair R., Pasqua T., Tota B., Angelone T., Cerra M.C., Nowosielski Y., Mätzler R., Gayen J.R., Trudeau V., Corti A., Helle K.B. (2017). Granin-derived peptides. Prog. Neurobiol..

[42] Zhang C., Rigbolt K., Petersen S.L., Biehl Rudkjær L.C., Schwahn U., Fernandez-Cachon M.L., Bossart M., Falkenhahn M., Theis S., Hübschle T., Schmidt T., Just Larsen P., Vrang N., Jelsing J. (2019). The preprohormone expression profile of enteroendocrine cells following Roux-en-Y gastric bypass in rats. Peptides.

[43] Samson W.K., Zhang J.V., Avsian-Kretchmer O., Cui K., Yosten G.L., Klein C., Lyu R.M., Wang Y.X., Chen X.Q., Yang J., Price C.J., Hoyda T.D., Ferguson A.V., Yuan X.B., Chang J.K., Hsueh A.J. (2008). Neuronostatin encoded by the somatostatin gene regulates neuronal, cardiovascular, and metabolic functions. J. Biol. Chem..

[44] Yosten G.L., Lyu R.M., Hsueh A.J., Avsian-Kretchmer O., Chang J.K., Tullock C.W., Dun S.L., Dun N., Samson W.K. (2013). A novel reproductive peptide, phoenixin. J. Neuroendocrinol..

[45] Wichmann A., Allahyar A., Greiner T.U., Plovier H., Lundén G., Larsson T., Drucker D.J., Delzenne N.M., Cani P.D., Bäckhed F. (2013). Microbial modulation of energy availability in the colon regulates intestinal transit. Cell Host Microbe.

[46] Agouni A., Owen C., Czopek A., Mody N., Delibegovic M. (2010). In vivo differential effects of fasting, re-feeding, insulin and insulin stimulation time course on insulin signaling pathway components in peripheral tissues. Biochem. Biophys. Res. Commun..

