# Supplementary material for: Peptidomics of enteroendocrine cells and characterisation of potential effects of a novel preprogastrin derived-peptide on glucose tolerance in lean mice
Source: Peptides. 2021 Jun;140:170532. doi: 10.1016/j.peptides.2021.170532 (PMC8121762; doi:10.1016/j.peptides.2021.170532)
Supplement: Supplementary file 1 [file mmc1.docx]

Supplementary material

| **Peptide** | **Route** | **Dose (mg/kg)** | **Bioavailability (%)** | **AUC (h*µg/mL)** | **Clearance (mL/h/kg)** |
| --- | --- | --- | --- | --- | --- |
| **ChgA 435-462a** | i.v. | 1 | NA | 0.362 | 2.76 |
| **ChgA 435-462a** | s.c. | 1 | 46.7 | 0.169 | 5.92 |
| **ChgA 435-462a** | s.c. | 3 | 50.4 | 0.547 | 5.48 |
| **Gast p59-79** | i.v. | 1 | NA | 0.503 | 19.9 |
| **Gast p59-79** | s.c. | 10 | 110 | 0.558 | 17.9 |
| **Sst 25-36** | s.c. | 10 | - | 0.0033 | 3030 |
| **Table S1**: Bioavailability, AUC and clearance of the ChgA 435-462a, Gast p59-79 and Sst 25-36 for each dose and route of administration. Sst 25-36 was administered i.v. at 1 mg/kg but was undetectable in plasma samples. i.v.= intravenous, s.c.=subcutaneous | | | | | |

For supplementary figures and supplementary tables S2 and S3 see accompanying documents.
