# Supplementary material for: Peptidomics of enteroendocrine cells and characterisation of potential effects of a novel preprogastrin derived-peptide on glucose tolerance in lean mice
Source: Peptides. 2021 Jun;140:170532. doi: 10.1016/j.peptides.2021.170532 (PMC8121762; doi:10.1016/j.peptides.2021.170532)
Supplement: Supplementary file 2 [file mmc2.pptx]

## Slide 1
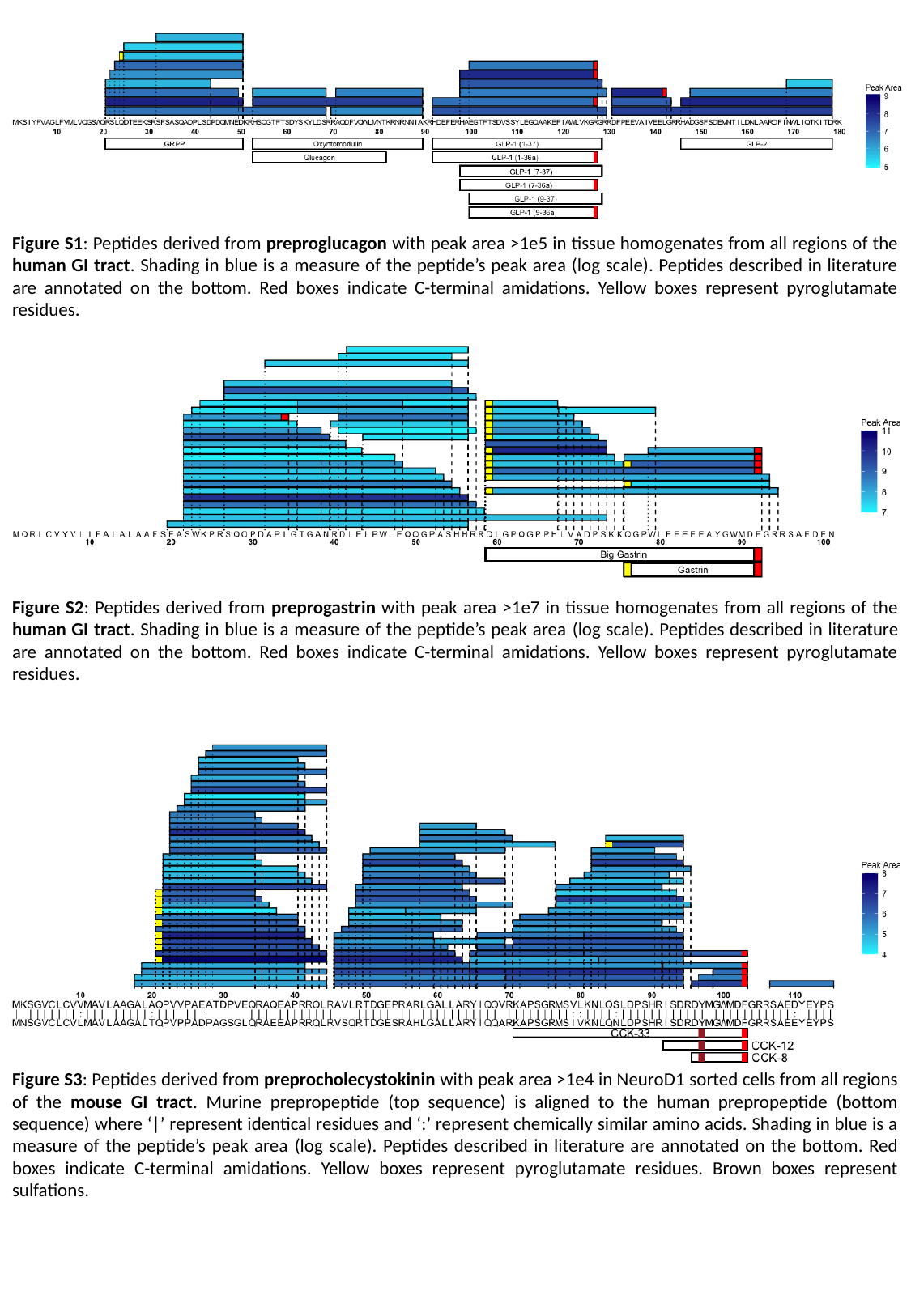

Figure S1: Peptides derived from preproglucagon with peak area >1e5 in tissue homogenates from all regions of the human GI tract. Shading in blue is a measure of the peptide’s peak area (log scale). Peptides described in literature are annotated on the bottom. Red boxes indicate C-terminal amidations. Yellow boxes represent pyroglutamate residues.
Figure S2: Peptides derived from preprogastrin with peak area >1e7 in tissue homogenates from all regions of the human GI tract. Shading in blue is a measure of the peptide’s peak area (log scale). Peptides described in literature are annotated on the bottom. Red boxes indicate C-terminal amidations. Yellow boxes represent pyroglutamate residues.
Figure S3: Peptides derived from preprocholecystokinin with peak area >1e4 in NeuroD1 sorted cells from all regions of the mouse GI tract. Murine prepropeptide (top sequence) is aligned to the human prepropeptide (bottom sequence) where ‘|’ represent identical residues and ‘:’ represent chemically similar amino acids. Shading in blue is a measure of the peptide’s peak area (log scale). Peptides described in literature are annotated on the bottom. Red boxes indicate C-terminal amidations. Yellow boxes represent pyroglutamate residues. Brown boxes represent sulfations.

## Slide 2
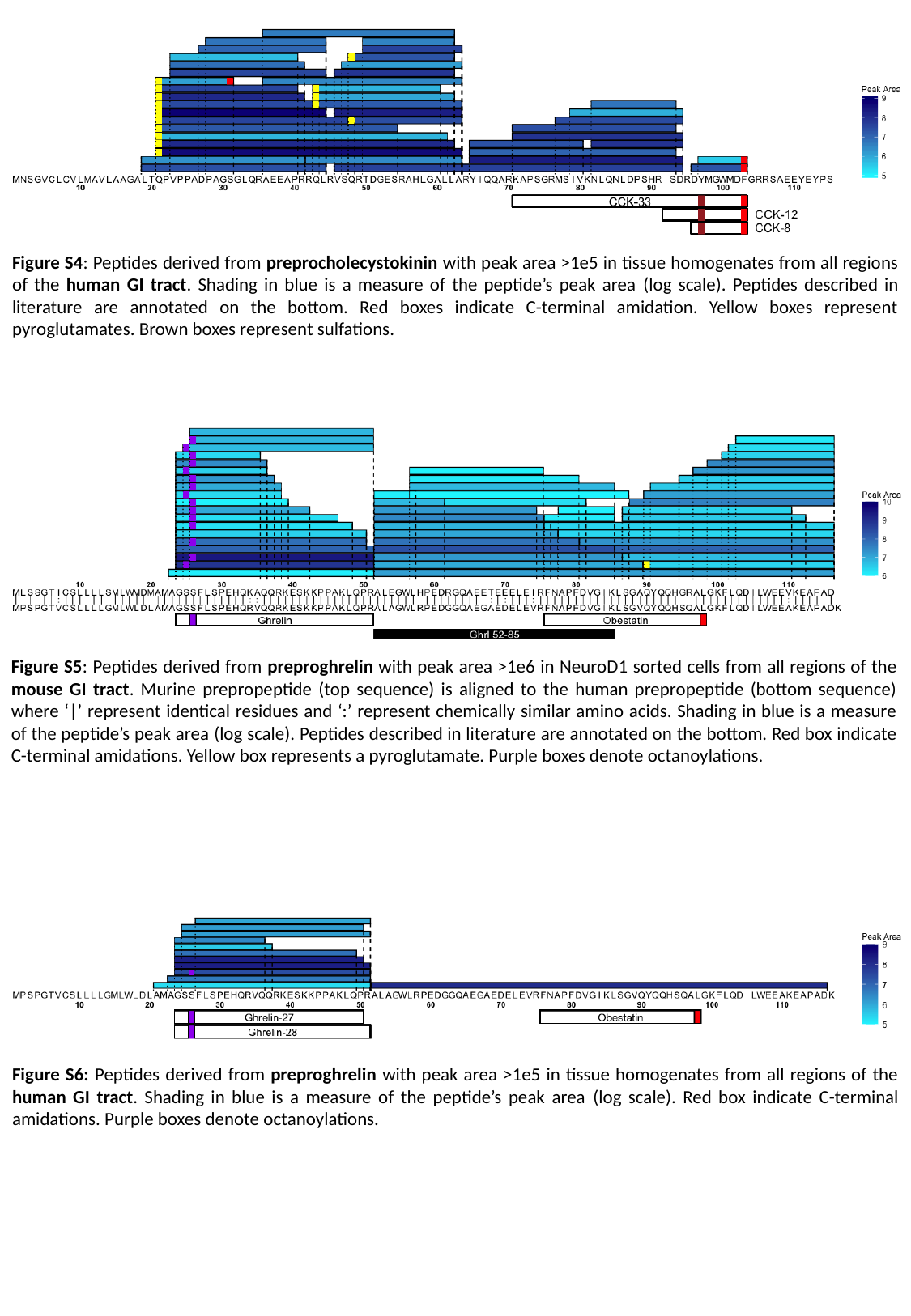

Figure S4: Peptides derived from preprocholecystokinin with peak area >1e5 in tissue homogenates from all regions of the human GI tract. Shading in blue is a measure of the peptide’s peak area (log scale). Peptides described in literature are annotated on the bottom. Red boxes indicate C-terminal amidation. Yellow boxes represent pyroglutamates. Brown boxes represent sulfations.
Figure S5: Peptides derived from preproghrelin with peak area >1e6 in NeuroD1 sorted cells from all regions of the mouse GI tract. Murine prepropeptide (top sequence) is aligned to the human prepropeptide (bottom sequence) where ‘|’ represent identical residues and ‘:’ represent chemically similar amino acids. Shading in blue is a measure of the peptide’s peak area (log scale). Peptides described in literature are annotated on the bottom. Red box indicate C-terminal amidations. Yellow box represents a pyroglutamate. Purple boxes denote octanoylations.
Figure S6: Peptides derived from preproghrelin with peak area >1e5 in tissue homogenates from all regions of the human GI tract. Shading in blue is a measure of the peptide’s peak area (log scale). Red box indicate C-terminal amidations. Purple boxes denote octanoylations.

## Slide 3
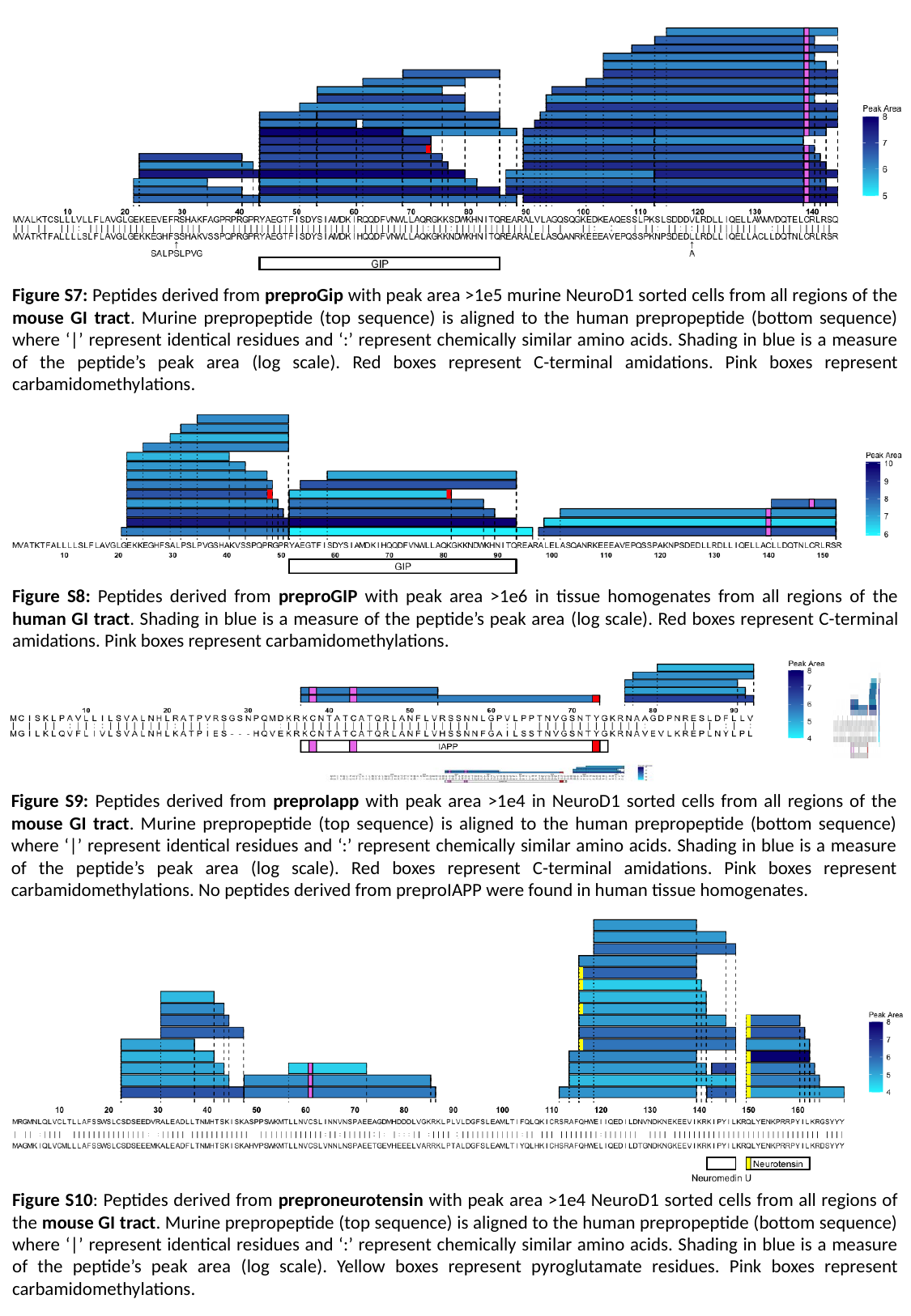

Figure S7: Peptides derived from preproGip with peak area >1e5 murine NeuroD1 sorted cells from all regions of the mouse GI tract. Murine prepropeptide (top sequence) is aligned to the human prepropeptide (bottom sequence) where ‘|’ represent identical residues and ‘:’ represent chemically similar amino acids. Shading in blue is a measure of the peptide’s peak area (log scale). Red boxes represent C-terminal amidations. Pink boxes represent carbamidomethylations.
Figure S8: Peptides derived from preproGIP with peak area >1e6 in tissue homogenates from all regions of the human GI tract. Shading in blue is a measure of the peptide’s peak area (log scale). Red boxes represent C-terminal amidations. Pink boxes represent carbamidomethylations.
Figure S9: Peptides derived from preproIapp with peak area >1e4 in NeuroD1 sorted cells from all regions of the mouse GI tract. Murine prepropeptide (top sequence) is aligned to the human prepropeptide (bottom sequence) where ‘|’ represent identical residues and ‘:’ represent chemically similar amino acids. Shading in blue is a measure of the peptide’s peak area (log scale). Red boxes represent C-terminal amidations. Pink boxes represent carbamidomethylations. No peptides derived from preproIAPP were found in human tissue homogenates.
Figure S10: Peptides derived from preproneurotensin with peak area >1e4 NeuroD1 sorted cells from all regions of the mouse GI tract. Murine prepropeptide (top sequence) is aligned to the human prepropeptide (bottom sequence) where ‘|’ represent identical residues and ‘:’ represent chemically similar amino acids. Shading in blue is a measure of the peptide’s peak area (log scale). Yellow boxes represent pyroglutamate residues. Pink boxes represent carbamidomethylations.

## Slide 4
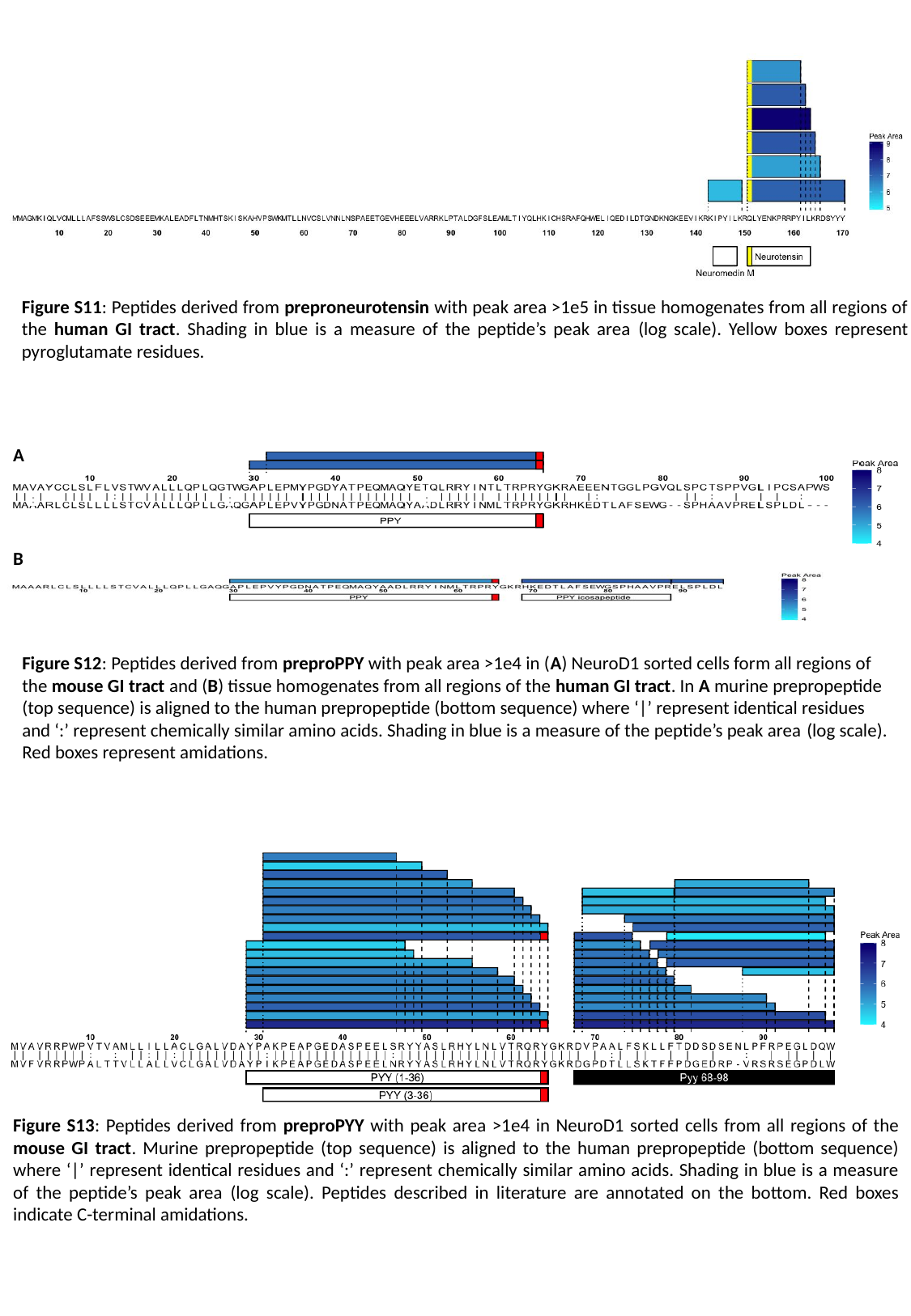

Figure S11: Peptides derived from preproneurotensin with peak area >1e5 in tissue homogenates from all regions of the human GI tract. Shading in blue is a measure of the peptide’s peak area (log scale). Yellow boxes represent pyroglutamate residues.
A
B
Figure S12: Peptides derived from preproPPY with peak area >1e4 in (A) NeuroD1 sorted cells form all regions of the mouse GI tract and (B) tissue homogenates from all regions of the human GI tract. In A murine prepropeptide (top sequence) is aligned to the human prepropeptide (bottom sequence) where ‘|’ represent identical residues and ‘:’ represent chemically similar amino acids. Shading in blue is a measure of the peptide’s peak area (log scale). Red boxes represent amidations.
Figure S13: Peptides derived from preproPYY with peak area >1e4 in NeuroD1 sorted cells from all regions of the mouse GI tract. Murine prepropeptide (top sequence) is aligned to the human prepropeptide (bottom sequence) where ‘|’ represent identical residues and ‘:’ represent chemically similar amino acids. Shading in blue is a measure of the peptide’s peak area (log scale). Peptides described in literature are annotated on the bottom. Red boxes indicate C-terminal amidations.

## Slide 5
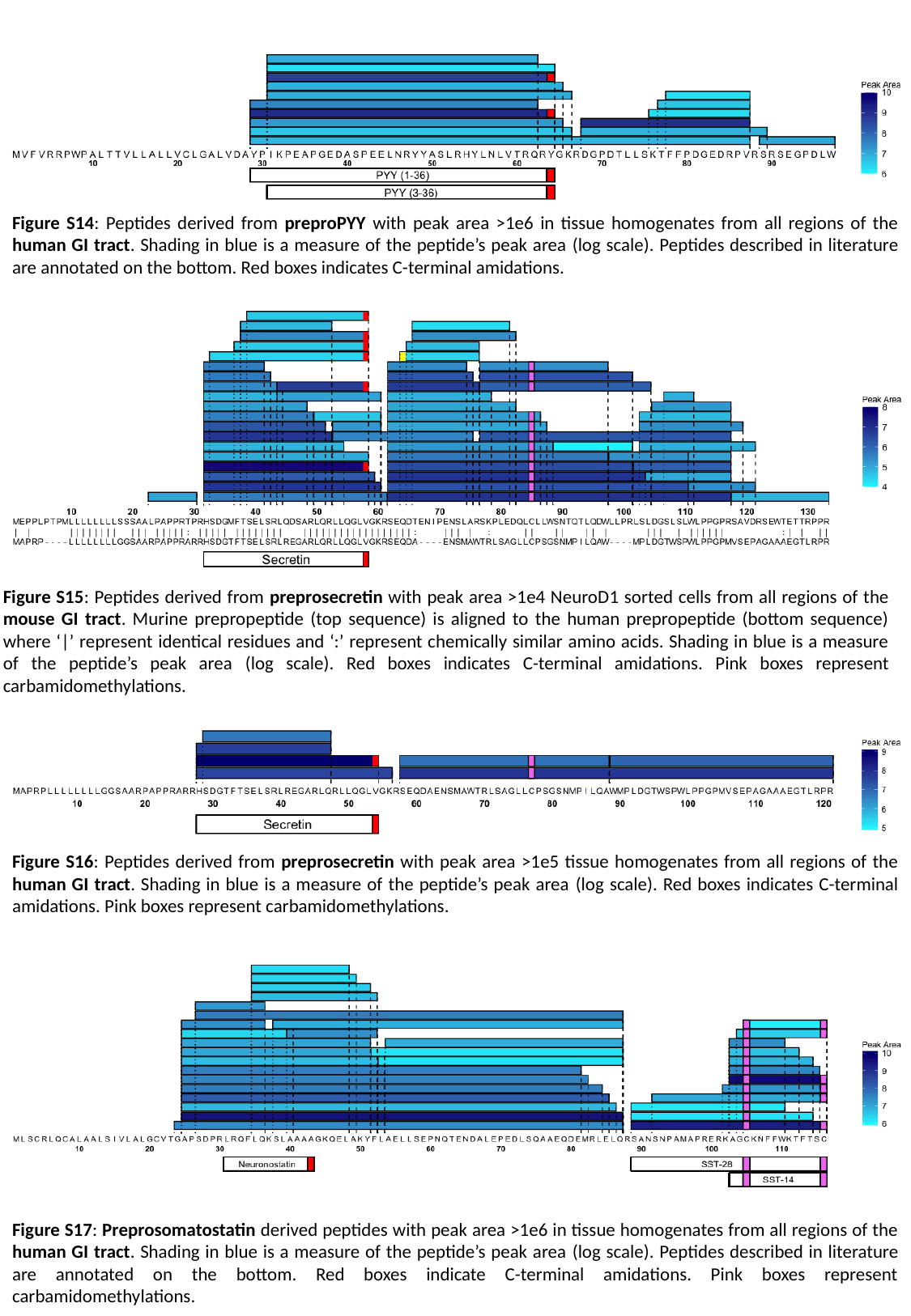

Figure S14: Peptides derived from preproPYY with peak area >1e6 in tissue homogenates from all regions of the human GI tract. Shading in blue is a measure of the peptide’s peak area (log scale). Peptides described in literature are annotated on the bottom. Red boxes indicates C-terminal amidations.
Figure S15: Peptides derived from preprosecretin with peak area >1e4 NeuroD1 sorted cells from all regions of the mouse GI tract. Murine prepropeptide (top sequence) is aligned to the human prepropeptide (bottom sequence) where ‘|’ represent identical residues and ‘:’ represent chemically similar amino acids. Shading in blue is a measure of the peptide’s peak area (log scale). Red boxes indicates C-terminal amidations. Pink boxes represent carbamidomethylations.
Figure S16: Peptides derived from preprosecretin with peak area >1e5 tissue homogenates from all regions of the human GI tract. Shading in blue is a measure of the peptide’s peak area (log scale). Red boxes indicates C-terminal amidations. Pink boxes represent carbamidomethylations.
Figure S17: Preprosomatostatin derived peptides with peak area >1e6 in tissue homogenates from all regions of the human GI tract. Shading in blue is a measure of the peptide’s peak area (log scale). Peptides described in literature are annotated on the bottom. Red boxes indicate C-terminal amidations. Pink boxes represent carbamidomethylations.

## Slide 6
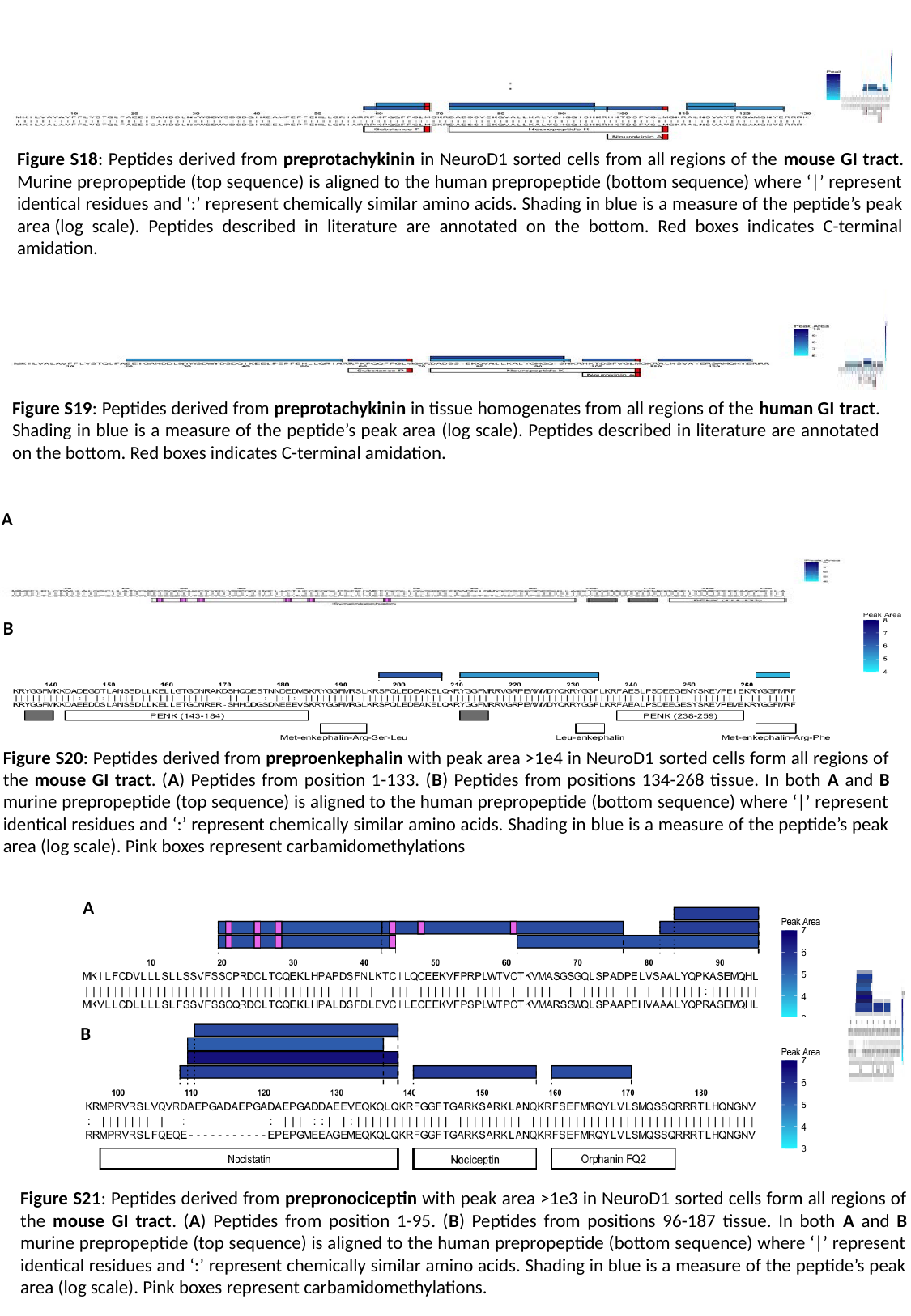

Figure S18: Peptides derived from preprotachykinin in NeuroD1 sorted cells from all regions of the mouse GI tract. Murine prepropeptide (top sequence) is aligned to the human prepropeptide (bottom sequence) where ‘|’ represent identical residues and ‘:’ represent chemically similar amino acids. Shading in blue is a measure of the peptide’s peak area (log scale). Peptides described in literature are annotated on the bottom. Red boxes indicates C-terminal amidation.
Figure S19: Peptides derived from preprotachykinin in tissue homogenates from all regions of the human GI tract. Shading in blue is a measure of the peptide’s peak area (log scale). Peptides described in literature are annotated on the bottom. Red boxes indicates C-terminal amidation.
A
B
Figure S20: Peptides derived from preproenkephalin with peak area >1e4 in NeuroD1 sorted cells form all regions of the mouse GI tract. (A) Peptides from position 1-133. (B) Peptides from positions 134-268 tissue. In both A and B murine prepropeptide (top sequence) is aligned to the human prepropeptide (bottom sequence) where ‘|’ represent identical residues and ‘:’ represent chemically similar amino acids. Shading in blue is a measure of the peptide’s peak area (log scale). Pink boxes represent carbamidomethylations
Figure S21: Peptides derived from prepronociceptin with peak area >1e3 in NeuroD1 sorted cells form all regions of the mouse GI tract. (A) Peptides from position 1-95. (B) Peptides from positions 96-187 tissue. In both A and B murine prepropeptide (top sequence) is aligned to the human prepropeptide (bottom sequence) where ‘|’ represent identical residues and ‘:’ represent chemically similar amino acids. Shading in blue is a measure of the peptide’s peak area (log scale). Pink boxes represent carbamidomethylations.
A
B

## Slide 7
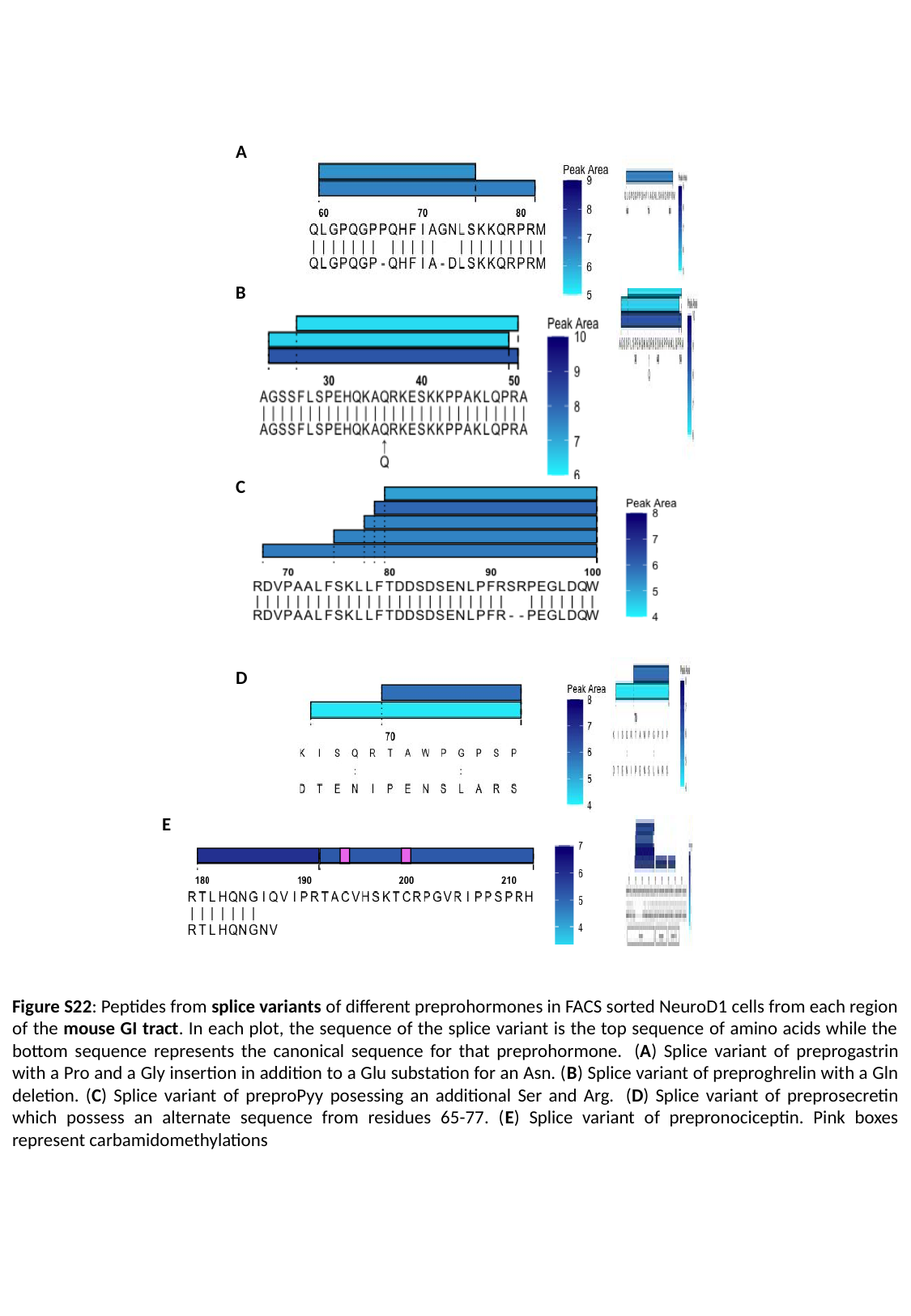

A
B
C
D
E
Figure S22: Peptides from splice variants of different preprohormones in FACS sorted NeuroD1 cells from each region of the mouse GI tract. In each plot, the sequence of the splice variant is the top sequence of amino acids while the bottom sequence represents the canonical sequence for that preprohormone.  (A) Splice variant of preprogastrin with a Pro and a Gly insertion in addition to a Glu substation for an Asn. (B) Splice variant of preproghrelin with a Gln deletion. (C) Splice variant of preproPyy posessing an additional Ser and Arg.  (D) Splice variant of preprosecretin which possess an alternate sequence from residues 65-77. (E) Splice variant of prepronociceptin. Pink boxes represent carbamidomethylations

## Slide 8
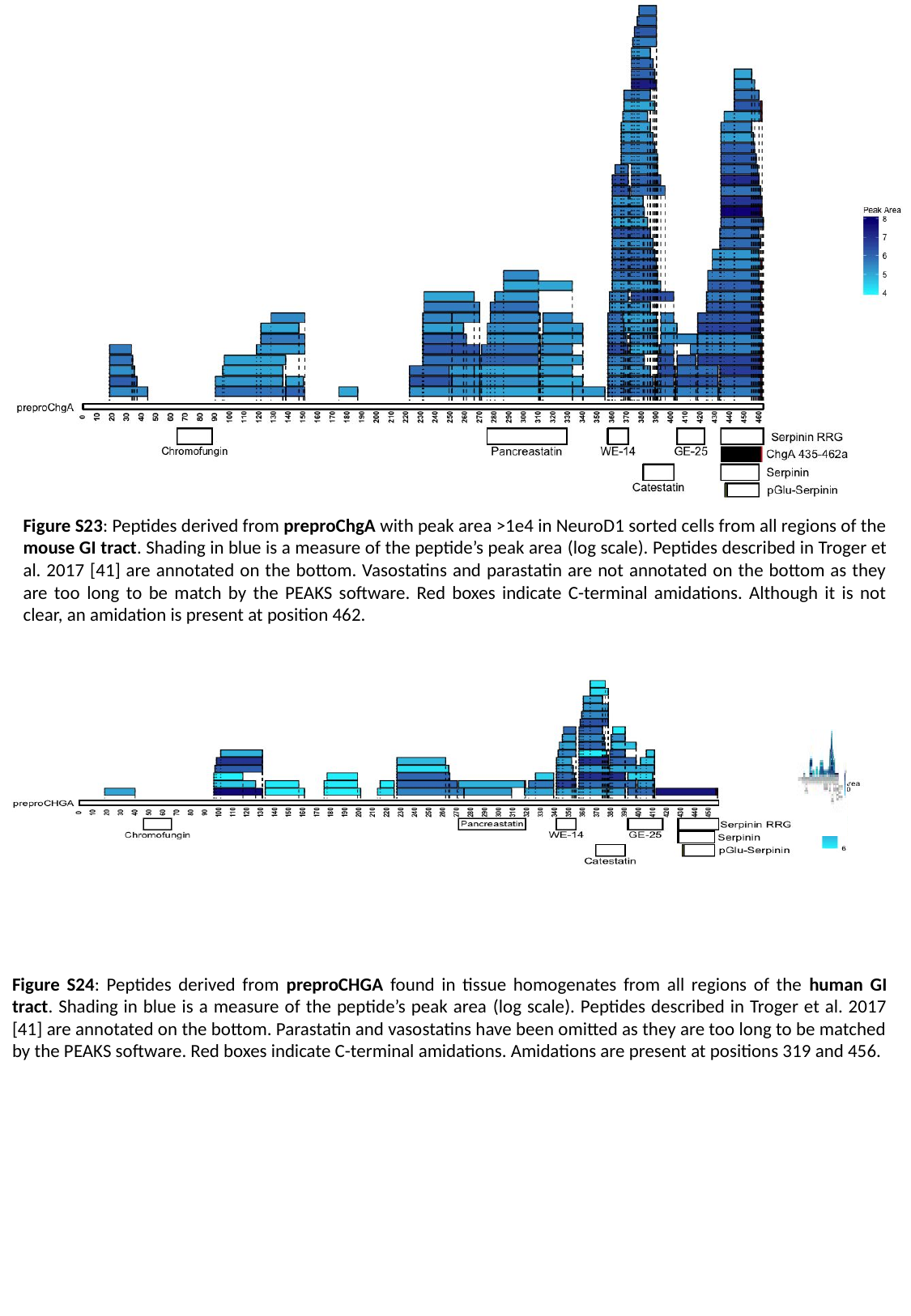

Figure S23: Peptides derived from preproChgA with peak area >1e4 in NeuroD1 sorted cells from all regions of the mouse GI tract. Shading in blue is a measure of the peptide’s peak area (log scale). Peptides described in Troger et al. 2017 [41] are annotated on the bottom. Vasostatins and parastatin are not annotated on the bottom as they are too long to be match by the PEAKS software. Red boxes indicate C-terminal amidations. Although it is not clear, an amidation is present at position 462.
Figure S24: Peptides derived from preproCHGA found in tissue homogenates from all regions of the human GI tract. Shading in blue is a measure of the peptide’s peak area (log scale). Peptides described in Troger et al. 2017 [41] are annotated on the bottom. Parastatin and vasostatins have been omitted as they are too long to be matched by the PEAKS software. Red boxes indicate C-terminal amidations. Amidations are present at positions 319 and 456.

## Slide 9
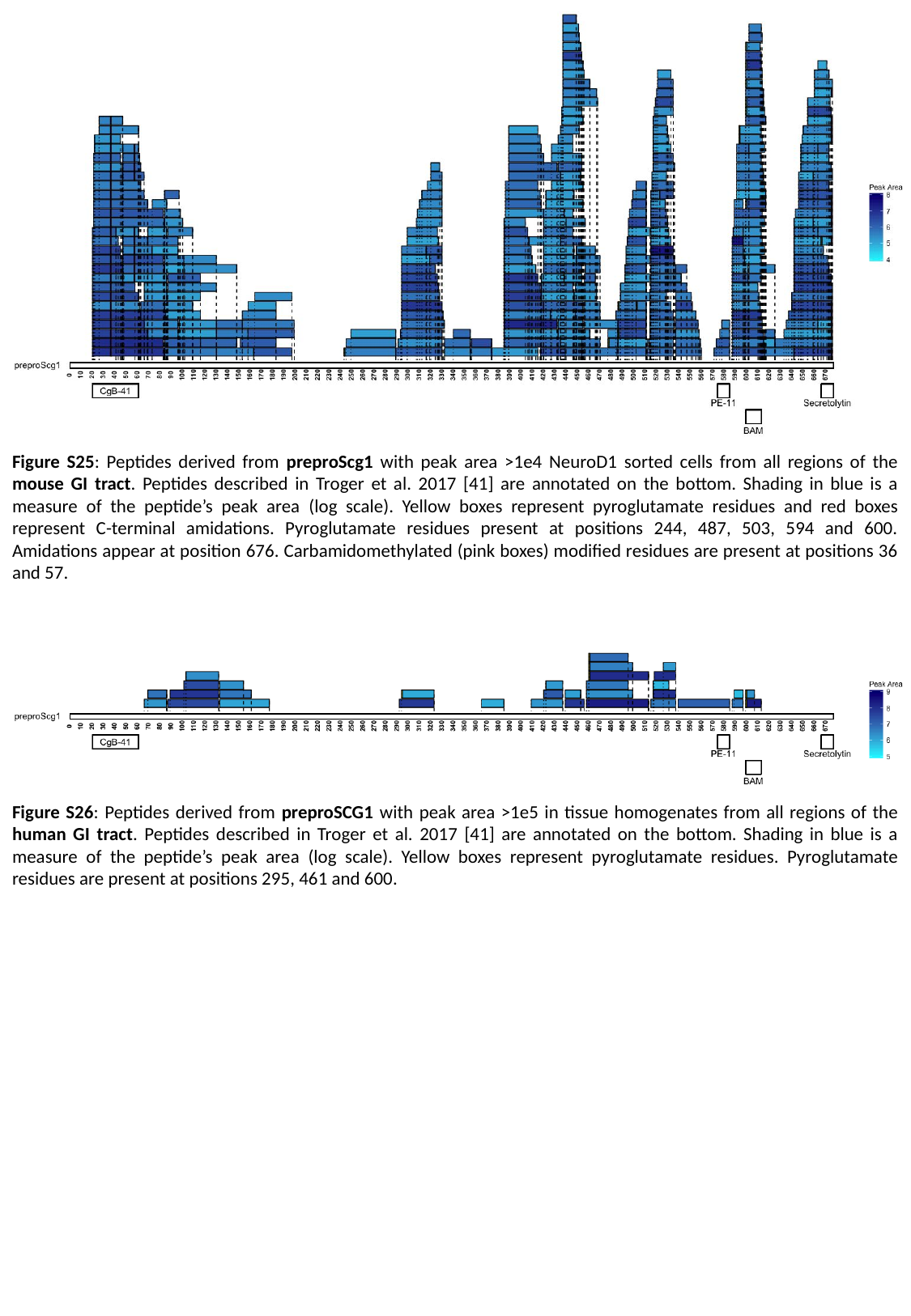

Figure S25: Peptides derived from preproScg1 with peak area >1e4 NeuroD1 sorted cells from all regions of the mouse GI tract. Peptides described in Troger et al. 2017 [41] are annotated on the bottom. Shading in blue is a measure of the peptide’s peak area (log scale). Yellow boxes represent pyroglutamate residues and red boxes represent C-terminal amidations. Pyroglutamate residues present at positions 244, 487, 503, 594 and 600. Amidations appear at position 676. Carbamidomethylated (pink boxes) modified residues are present at positions 36 and 57.
Figure S26: Peptides derived from preproSCG1 with peak area >1e5 in tissue homogenates from all regions of the human GI tract. Peptides described in Troger et al. 2017 [41] are annotated on the bottom. Shading in blue is a measure of the peptide’s peak area (log scale). Yellow boxes represent pyroglutamate residues. Pyroglutamate residues are present at positions 295, 461 and 600.

## Slide 10
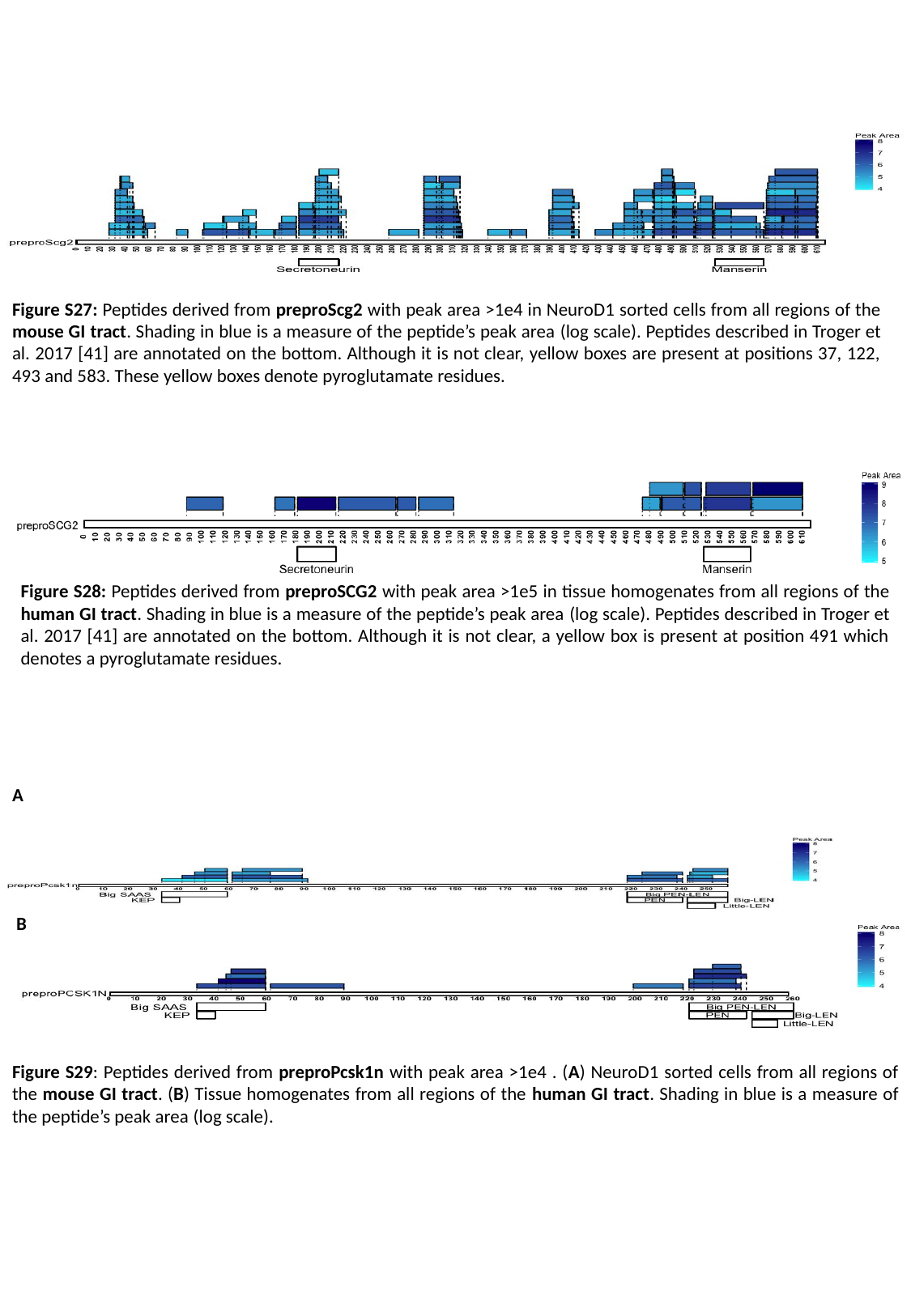

Figure S27: Peptides derived from preproScg2 with peak area >1e4 in NeuroD1 sorted cells from all regions of the mouse GI tract. Shading in blue is a measure of the peptide’s peak area (log scale). Peptides described in Troger et al. 2017 [41] are annotated on the bottom. Although it is not clear, yellow boxes are present at positions 37, 122, 493 and 583. These yellow boxes denote pyroglutamate residues.
Figure S28: Peptides derived from preproSCG2 with peak area >1e5 in tissue homogenates from all regions of the human GI tract. Shading in blue is a measure of the peptide’s peak area (log scale). Peptides described in Troger et al. 2017 [41] are annotated on the bottom. Although it is not clear, a yellow box is present at position 491 which denotes a pyroglutamate residues.
A
B
Figure S29: Peptides derived from preproPcsk1n with peak area >1e4 . (A) NeuroD1 sorted cells from all regions of the mouse GI tract. (B) Tissue homogenates from all regions of the human GI tract. Shading in blue is a measure of the peptide’s peak area (log scale).

## Slide 11
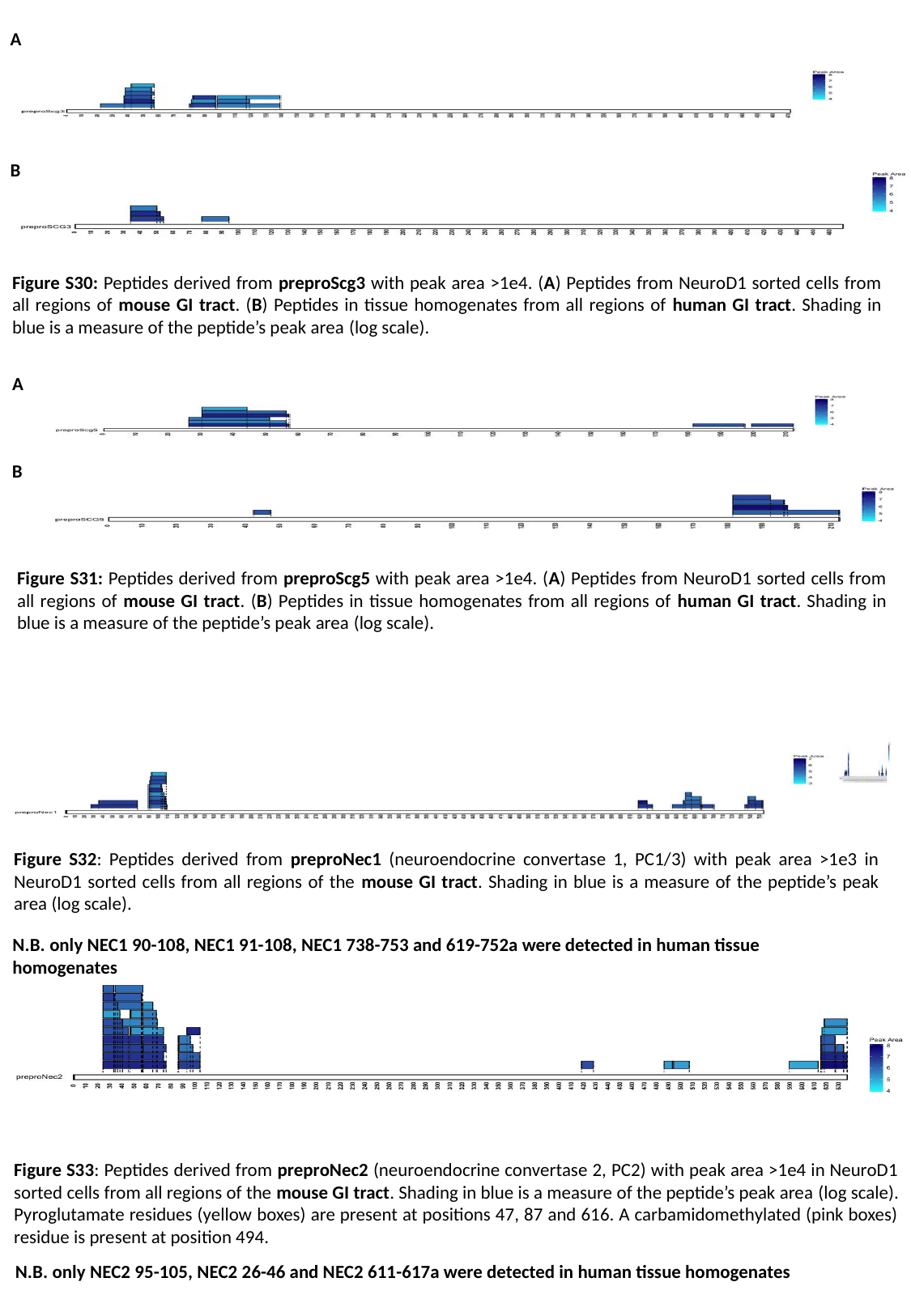

A
B
Figure S30: Peptides derived from preproScg3 with peak area >1e4. (A) Peptides from NeuroD1 sorted cells from all regions of mouse GI tract. (B) Peptides in tissue homogenates from all regions of human GI tract. Shading in blue is a measure of the peptide’s peak area (log scale).
A
B
Figure S31: Peptides derived from preproScg5 with peak area >1e4. (A) Peptides from NeuroD1 sorted cells from all regions of mouse GI tract. (B) Peptides in tissue homogenates from all regions of human GI tract. Shading in blue is a measure of the peptide’s peak area (log scale).
Figure S32: Peptides derived from preproNec1 (neuroendocrine convertase 1, PC1/3) with peak area >1e3 in NeuroD1 sorted cells from all regions of the mouse GI tract. Shading in blue is a measure of the peptide’s peak area (log scale).
N.B. only NEC1 90-108, NEC1 91-108, NEC1 738-753 and 619-752a were detected in human tissue homogenates
Figure S33: Peptides derived from preproNec2 (neuroendocrine convertase 2, PC2) with peak area >1e4 in NeuroD1 sorted cells from all regions of the mouse GI tract. Shading in blue is a measure of the peptide’s peak area (log scale). Pyroglutamate residues (yellow boxes) are present at positions 47, 87 and 616. A carbamidomethylated (pink boxes) residue is present at position 494.
N.B. only NEC2 95-105, NEC2 26-46 and NEC2 611-617a were detected in human tissue homogenates
